# Higher-order statistics for constructing centered edge functional connectivity

**DOI:** 10.1162/NETN.a.570

**Published:** 2026-07-27

**Authors:** Junting Wang, Youngheun Jo, Junwei Lu, Richard Betzel, Ji Zhu, Kean Ming Tan

**Affiliations:** Department of Statistics, University of Michigan, Ann Arbor, MI, USA; Department of Psychiatry, University of Michigan, Ann Arbor, MI, USA; Department of Biostatistics, Harvard University, Boston, MA, USA; Department of Neuroscience, University of Minnesota, Minneapolis, MN, USA

**Keywords:** Brain connectivity networks, Covariance matrix, Edge functional connectivity, fMRI

## Abstract

Functional connectivity is often constructed to understand functional organizations in neural systems, where the brain regions and their pairwise interactions are viewed as nodes and edges, respectively. In practice, functional connectivity is commonly estimated via the correlation of pairs of brain regions. One limitation is that the correlation coefficient captures only the pairwise linear dependence relationship between pairs of nodes and may fail to capture complex higher-order relationships. Recently, a novel concept known as edge-centric functional connectivity (eFC) has been introduced to measure interactions between pairs of edges based on the cofluctuation of two nodal time series, offering a new perspective for understanding brain networks. Nevertheless, eFC considers the absolute levels of edge time series, that is, their mean values, in estimation. If the parameter of interest is the covariation between a pair of edges, incorporating mean values of edge time series can introduce bias or deviation, resulting in a skewed estimation. In this manuscript, we propose an alternative approach to estimate the unbiased covariation between pairs of edges, termed centered edge functional connectivity (ceFC), with theoretical foundations. We demonstrate that the proposed estimator is consistent with a sufficient sample size or number of time frames. Additionally, we develop a multiple hypothesis testing framework with a controlled false discovery rate to evaluate the strength of the unbiased covariation among edges. Furthermore, we employ thresholding to obtain a thresholded estimator that has been shown to converge to the true ceFC matrix in high-dimensional settings in which the number of nodes is much larger than the number of samples or time frames. We validate the finite sample performance of the proposed methods via numerical studies and a data application using the Midnight Scan Club dataset.

## Author Summary

Functional connectivity typically examines the correlations between pairs of brain nodes, which only capture pairwise linear dependence. Recently, the edge-centric functional connectivity (eFC) method has shifted the focus toward the relationships between pairs of edges. While promising, this current method can introduce statistical biases that skew our understanding of these higher-order interactions. In this study, we propose centered edge functional connectivity (ceFC), a framework designed to provide an unbiased view of how brain edges covary. By establishing a rigorous statistical foundation and an inference framework to identify significant interactions, our method ensures reliable results even with a limited sample size. We show that our approach can help identify more stable overlapping brain community structures, providing a new perspective on brain organization.

## INTRODUCTION

Recent technological advancements, such as functional magnetic resonance imaging (fMRI), have significantly advanced our understanding of brain function through cost-efficient and noninvasive (albeit indirect) acquisition measures of brain activity. fMRI detects changes in spontaneous and task-free BOLD fluctuations under specific tasks or at rest, yielding an indirect measure of brain activity over time (Kwong et al., 1992; Ogawa, Lee, Kay, & Tank, 1990). Using these spatiotemporal data, researchers can calculate the temporal pairwise interactions among neural elements, often estimated through pairwise regional correlations, as a measure of functional connectivity (Aertsen, Gerstein, Habib, & Palm, 1989; Friston, 1994; Friston, Frith, Liddle, & Frackowiak, 1993). Numerous studies have applied functional connectivity in brain networks (Finn et al., 2015; Power et al., 2011), shedding light on the brain’s functional organization and identifying putative biomarkers for a range of diseases (Greicius, Krasnow, Reiss, & Menon, 2003; Wen et al., 2018; Yeo et al., 2011), including but not limited to autism spectrum disorders (Monk et al., 2009; Weng et al., 2010), Alzheimer’s disease (Supekar, Menon, Rubin, Musen, & Greicius, 2008), major depressive disorder (Kaiser, Andrews-Hanna, Wager, & Pizzagalli, 2015; Yan et al., 2019), and mild cognitive impairment (Wang et al., 2020). In addition to such group-wise differences in functional connectivity between healthy individuals and patients, the existing literature also reveals heterogeneous patterns in brain connectivity since each individual is unique (Mueller et al., 2013), holding the potential for personalized treatment (Finn & Constable, 2016; Finn et al., 2015).

Despite the insights gained from conventional static functional connectivity, previous studies suggest that there may be higher-order interactions involved in functional connectivity (Gatica et al., 2021; Glickfeld & Olsen, 2017; Luppi et al., 2022), which may be obscured when using field-standard node-based approaches. For instance, higher-order estimates of functional connectivity may provide a more detailed understanding of functional brain networks through pervasive, nontrivial overlapping community organizations (Beckmann, DeLuca, Devlin, & Smith, 2005; Palla, Derényi, Farkas, & Vicsek, 2005; van den Heuvel & Sporns, 2013; Yeo, Krienen, Chee, & Buckner, 2014). Various evidence has indicated that brain regions participate in multiple functional roles (Ackerman, 1992; Hove & Martinez, 2024; Rishel, Huang, & Freedman, 2013), highlighting the importance of studying overlapping communities within brain networks, which can be trivially detected using an edge-centric approach within functional brain networks. In addition, higher-order connectivity in brain studies has been shown to provide valuable insights into various types of disorders, including but not limited to cognitive and neurological disorders, which may assist in the early detection and diagnosis of diseases (Chen et al., 2016; Plis et al., 2014; Zhang, Zhang, Chen, Lee, & Shen, 2017).

Recently, a novel concept of edge-centric functional connectivity (eFC), which examines connectivity between pairs of edges, was introduced in Faskowitz, Esfahlani, Jo, Sporns, and Betzel (2020). This edge-centric framework studies the cofluctuations in the BOLD signals between pairs of edges and has been applied in brain networks, revealing a diverse community structure in which brain regions can be grouped into multiple communities based on edge clusters (Faskowitz et al., 2020). The identification of overlapping communities by edge connectivity provides a deeper understanding of brain organization (Chumin et al., 2022), where at least two edge communities can link all brain systems (Jo, Esfahlani, et al., 2021). Recently, the edge-centric framework has been found to provide another perspective for understanding structural and functional features of brain networks (Esfahlani et al., 2020; Liu et al., 2022; Pope, Fukushima, Betzel, & Sporns, 2021; Wen et al., 2022), identifying individual differences (Jo, Faskowitz, Esfahlani, Sporns, & Betzel, 2021; Ville, Farouj, Preti, Liégeois, & Amico, 2021), serving as biomarkers for various disorders (Dai et al., 2023; Sun, Wang, & Zhang, 2023; Yang et al., 2023; Zamani Esfahlani et al., 2022), and uncovering the mechanism underlying therapeutic efficacy (Khambhati, Shafi, Rao, & Chang, 2021).

The proposed eFC measures the strength of connectivity between edge pairs based on the cofluctuation of edge time series while also taking the absolute levels of edge time series, that is, their mean values, into consideration. However, including the mean values of edge time series may bias the estimation of the association between edge pairs when the parameter of interest is their covariation. This can occur because the mean values can inflate or deflate the perceived strength of relationships between edges, leading to a skewed measurement and potentially obscuring the true underlying covariation between edges. To address this issue, the present study aims to provide an unbiased estimation of the covariation between edge pairs, denoted as centered edge functional connectivity (ceFC), and to investigate the statistical properties of its estimators. Each brain parcel is treated as a variable in our analysis. For a single subject, one intuitive estimation of ceFC, denoted as **Θ**, can be achieved using the sample moment estimator, which consists of the empirical estimators of the fourth-order and second-order moments by definition. Under the assumption of sub-Gaussian variables, we study the rate of convergence of the sample moment estimator $\hat{\Theta }$ under the Frobenius norm. Additionally, we develop a multiple hypothesis testing framework to examine the nonzero elements of the ceFC matrix while controlling the false discovery rate (FDR). Considering that different edge pairs can be dependent, our inference framework employs the Benjamini–Yekutieli procedure, a method designed to control FDR when accounting for dependencies between pairs of statistical tests (Benjamini & Yekutieli, 2001).

An important consideration for creating a reliable sample moment estimator is ensuring that the number of time frames is sufficient relative to the number of variables, that is, the number of brain regions. In fMRI studies, there are multiple schemes to parcellate human brains into brain regions, or regions of interest, where the common choices range from 50 up to 1,000 parcels (Glasser et al., 2016; Gordon et al., 2016; Power et al., 2011; Schaefer et al., 2018; Yeo et al., 2011). Different brain parcellations can impact functional connectivity magnitudes and generate differences among individuals (Bryce et al., 2021), while the number of time frames can be relatively limited in comparison with the high level of brain parcellations, particularly during specific tasks (Van Essen et al., 2013). It can be challenging when the number of brain areas is comparable with or even greater than the number of time frames during scan sessions, as the sample estimator for covariance becomes singular, whereas the actual covariance matrix is always positive definite.

In high-dimensional settings, it has been shown that the sample covariance typically does not perform well (Johnstone, 2001; Johnstone & Lu, 2009; Marčenko & Pastur, 1967), in which the estimation error can fail to converge to zero. To address this issue, significant effort has been devoted to developing alternative estimators. There is a rich literature on estimating large covariance matrices through banding or tapering (Bickel & Levina, 2008b; Bien, Bunea, & Xiao, 2016; Cai, Zhang, & Zhou, 2010; Yi & Zou, 2013), thresholding (Bickel & Levina, 2008a; T. Cai & Liu, 2011; Karoui, 2008; Rothman, Levina, & Zhu, 2009), and penalization (Lam & Fan, 2009; Ravikumar, Wainwright, Raskutti, & Yu, 2011). These techniques have been applied in various domains (Asai, Gupta, & McAleer, 2020; Fan & Liu, 2013; Markon, 2010; Xu & Lindquist, 2015), including neuroscience. For example, people have employed thresholding operators to eliminate weak connections in brain networks and reveal distinct features of brain networks (De Vico Fallani, Latora, & Chavez, 2017; Garrison, Scheinost, Finn, Shen, & Constable, 2015; Nicolini, Forcellini, Minati, & Bifone, 2020; Stam, 2004). In light of this, we adopt the thresholding operator **T***_τ_* to truncate values in the sample moment estimator below a certain threshold *τ* to 0 as the thresholded estimator for a class of sparse ceFC matrices.

In this study, we aim to explore the unbiased estimators of covariation among edges under both general scenarios with sufficient sample sizes and the high-dimensional setting in which the sample sizes is smaller than the number of variables. We demonstrate that both types of estimators for centered eFC—the sample moment estimator and the proposed thresholded estimator—provide consistent results through statistical rates of convergence and simulation studies. Building on the sample moment estimator, we construct a multiple hypothesis testing framework to examine the sparsity of the ceFC matrix and demonstrate that this inference framework achieves asymptotic control of FDR. We also apply the proposed estimators to the Midnight Scan Club (MSC) dataset (Gordon et al., 2017) and investigate the underlying overlapping structures through edge clustering. By evaluating the clustering results from three types of estimators, we show that the FDR-controlled and thresholded estimators provide relatively more stable clustering outcomes compared with those based on the sample moment estimator.

## RESULTS

The primary goal of our study is to introduce and validate an unbiased framework for connectivity between edges. We begin by briefly defining the key concepts underlying our analysis before presenting the main theoretical and empirical results.

At its core, traditional FC measures the statistical dependence between pairs of brain regions (e.g., correlation; Aertsen et al., 1989; Friston, 1994; Friston et al., 1993). A more recent concept, eFC, extends this idea to a higher order by quantifying the cofluctuation between pairs of edges themselves (Faskowitz et al., 2020). An edge time series can be constructed, for instance, by taking the element-wise product of the time series of two connected nodes. The original eFC is then calculated as a scaled measure of the cofluctuation between two such edge time series (Faskowitz et al., 2020).

A critical limitation of the eFC formulation is its sensitivity to the mean of the edge time series (i.e., the static functional connectivity). This can introduce a sample-specific bias, potentially impacting the reproducibility of scientific findings. To address this, we propose ceFC, which is mathematically defined as the unbiased covariation between two edge time series after their means have been removed. By design, ceFC isolates the pure dynamic cofluctuation between edges from the confounding influence of static connectivity. For a formal definition for ceFC, please refer to the ceFC section.

In this section, we evaluate two distinct estimators for ceFC. We first examine a straightforward sample moment estimator, which is analogous to the original eFC but applied to mean-centered data. We will demonstrate its statistical limitations in high-dimensional settings, that is, when the number of time frames is limited compared with the number of parcels. We then introduce and evaluate a more sophisticated thresholded estimator, designed to provide consistent and robust estimates even when the number of brain parcels is large relative to the number of time frames. Next, we present a multiple hypothesis testing framework to simultaneously evaluate the strength of each edge pair covariation, demonstrating that this inference procedure effectively controls the FDR. Finally, we apply this unbiased framework to identify and analyze overlapping functional brain communities on MSC dataset.

### Consistency Results for Sample Moment and Thresholded Estimators

In this section, we show that the sample moment estimator converges to the true ceFC matrix within a bound as a function of $\sqrt{N^{4}\log^{4}\left({TN}^{4}\right)/T}$, providing a consistent estimate when the sample size (or number of time frames) *T* is sufficiently large, specifically with *T* much larger than *N*^4^. We also extend our discussion to the high-dimensional setting with a limited number of time frames *T* compared with the number of brain parcels *N*, in which the thresholded estimator provides a consistent estimate when the true ceFC is sparse. In the following sections, we will present theoretical results for the convergence rates of the two types of estimators and evaluate their estimation performance through simulation studies.

#### Theoretical results for sample moment estimation.

We first study the performance of the sample moment estimator (Equation 6) in terms of a concentration inequality, providing a mathematical upper bound for the probability of its deviation from the true ceFC value with sufficient sample size. The formal definition can be found in the Sample Estimation of ceFC section. As the sample moment estimator $\hat{\Theta }=\left[{\hat{\Theta }}_{jk,st}\right]$ and the true ceFC **Θ** = [Θ_*jk*,*st*_] are both matrices, we measure the estimation error under the Frobenius norm, which is the sum of squared error of each *jk*, *st* pair

$${\left\|\hat{\Theta }-\Theta \right\|}_{F}=\sqrt{\sum_{jk,st}{\left(\Theta_{jk,st}-{\hat{\Theta }}_{jk,st}\right)}^{2}}.$$

Under the sub-Gaussian condition in the Null Hypothesis section with the Frobenius norm, the statistical rate, which describes the deviation from expectation of the sample moment estimator in Equation 6, takes the form

$${\left\|\hat{\Theta }-\Theta \right\|}_{F}^{2}\leq {CN}^{4}\frac{\log^{4}\left({TN}^{4}\right)}{T},$$ (1)

for some sufficiently large constant *C* that does not depend on *T* and *N*, with probability at least 1 − *T*^−1^. The detailed proof with technical lemmas can be found in the Supporting Information section. We can see that with a fixed number of parcels *N*, the estimation error measured under the Frobenius norm approaches zero with increasing sample size *T*. This convergent error demonstrates that our estimator is consistent and becomes increasingly accurate as more data are collected, ensuring reliable estimation of the true ceFC matrix in practical applications.

#### Theoretical results for thresholded estimation.

Next, we consider the scenario where the number of time frames *T* is smaller than the number of brain parcels *N*. In this situation, the estimation error for the sample moment estimator, which is upper bounded by $N^{2}\log^{2}\left({TN}^{4}\right)/\sqrt{T}$ under the Frobenius norm as shown in Equation 1, fails to converge to zero. This high-dimensional scenario presents a fundamental statistical challenge: With limited time frames, many of the observed weak (low-weight) connections are statistically indistinguishable from random noise or sampling error. It becomes infeasible to reliably estimate the numerous parameters in the ceFC matrix without additional structural constraints.

One common strategy to overcome inconsistent estimates in such a high-dimensional setting is to employ a thresholding operator, which has been widely adopted in the existing literature to achieve consistent estimation of large covariance matrices with *N* > *T* (Bickel & Levina, 2008a; T. Cai & Liu, 2011; Karoui, 2008; Rothman et al., 2009). This thresholding technique is often constructed based on structural assumptions that involve the sparsity of the target matrix and has been applied in various disciplines (Asai et al., 2020; Fan & Liu, 2013; Markon, 2010; Xu & Lindquist, 2015), including neuroscience (Buchanan et al., 2020; de Reus & van den Heuvel, 2013; Honey, Kötter, Breakspear, & Sporns, 2007).

Considering a class of sparse ceFC matrices, our proposed thresholded estimator applies the thresholding operator to the sample moment estimator with a chosen threshold *τ*, denoted as **T**_*τ*_($\hat{\Theta }$). That is, we truncate the entries with absolute values less than *τ* in the sample moment estimator $\hat{\Theta }$ to zero. The formal definition is stated in the Thresholded Estimator of ceFC section. This technique has been frequently employed in brain studies to eliminate spurious connections under the assumption that the likelihood of a connection being spurious decays monotonically with its weight, revealing features of brain networks (De Vico Fallani et al., 2017; Garrison et al., 2015; Nicolini et al., 2020; Stam, 2004).

Similar to the study of the sample moment estimator, under the sub-Gaussian assumption, we show that uniformly over the class of sparse ceFC matrices $U_{\tau }\left(m\right)$, where *m* represents the maximum number of nonzero entries in the true ceFC matrix, if we choose $\tau =C\sqrt{\log^{4}\left({TN}^{4}\right)/T}$, we have

$${\left\|T_{\tau }\left(\hat{\Theta }\right)-\Theta \right\|}_{F}^{2}\leq {mC}^{2}\frac{\log^{4}\left({TN}^{4}\right)}{T},$$ (2)

for some constant *C* with probability at least 1 − *T*^−1^. The derivation can be found in the Supporting Information section. With this upper bound, even if *N* > *T*, the estimation error for the thresholded estimator can still converge to zero, especially with sufficiently small *m*, which denotes the number of nonzero entries in the true ceFC matrix. Together, these observations suggest that we expect this thresholded estimator to converge to the true ceFC given long enough scans with sufficiently sparse connectivity among edge pairs.

#### Simulation results for estimation.

In this section, we perform extensive numerical studies to assess the performance of the sample moment estimator and the thresholded estimator for ceFC under different scenarios. We generate data under a multivariate Gaussian distribution that preserves the average correlation from the MSC data (Gordon et al., 2017), consisting of fMRI scans from 10 subjects, comprising 5 male and 5 female participants. We consider the set of brain parcels with *N* = 200. To illustrate a scenario with a smaller number of variables, a straightforward situation with sufficient samples, we randomly select 25% of the brain parcels from each system, resulting in a subset of *N* = 50 parcels. The sample size *T* depends on the number of time frames in the MSC scans, in which the volumes are acquired approximately every 2.2 s. See the Simulation Settings for Estimation Evaluation section for the detailed settings. With Gaussian variables, the true ceFC matrix can be obtained by Isserlis’s theorem (Isserlis, 1918). The proposed thresholded estimator involves selecting a tuning parameter *τ* > 0. As suggested by the consistency result (Equation 2) in the previous section, we set $\tau =C\cdot \sqrt{\log^{4}\left({TN}^{4}\right)/T}$ with *C* ∈ {0.002, 0.005, 0.01} for some weak to moderate controls under the simulation setting.

**Figure 1.**
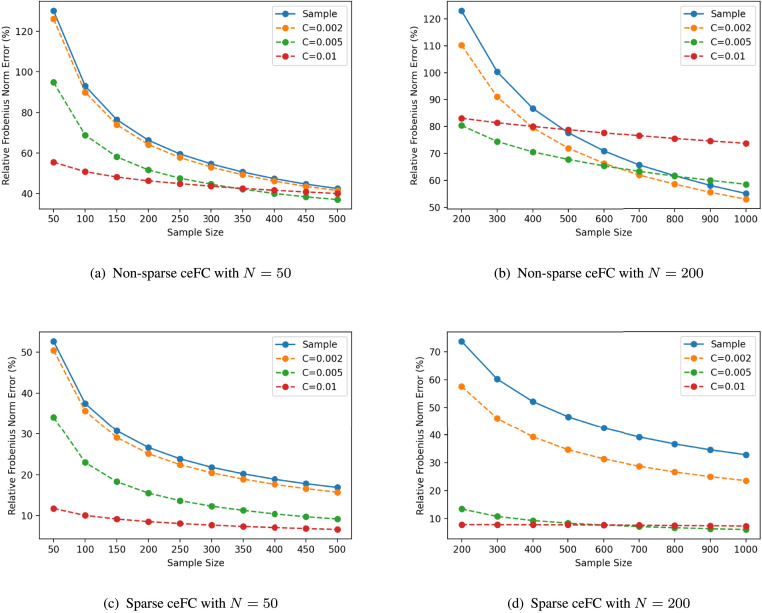
Relative Frobenius norm estimation error for the nonsparse (top) and sparse (bottom) ceFC matrix with the number of brain parcels set to *N* = 50 (left) or *N* = 200 (right). The colored lines correspond to the proposed sample moment estimator (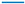), the thresholded estimators using the tuning parameter *C* = 0.002 (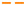), *C* = 0.005 (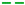), and *C* = 0.01 (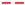). The sample size *T* (number of time frames), number of brain parcels *N*, and tuning parameter *C* determine the thresholding *τ* through $\tau =C\sqrt{\log^{4}\left({TN}^{4}\right)/T}$ for the thresholded estimator.

Building upon the preceding theoretical analysis, we quantify the estimation error between the estimated matrix and the true ceFC matrix under the Frobenius norm for both methods. In Figure 1, we summarize the relative average estimation error under the Frobenius norm across 100 independent replications for the nonsparse and sparse scenarios, respectively. From Figure 1, we observe that estimation errors for both nonthresholded and thresholded estimators decrease as we increase the number of time frames *T*. Our results also suggest that the thresholded estimator can outperform the sample moment estimator when the signal-to-noise ratio is small (i.e., when *T* is small), even when the true ceFC matrix is dense. Under the scenario in which the ceFC matrix is sparse, we see that the proposed thresholded estimator consistently outperforms the sample moment estimator for all truncation parameter choices across varying *C*.

**Figure 2.**
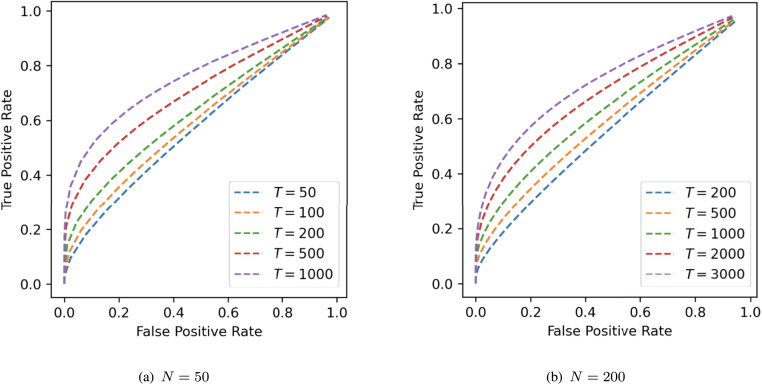
The TPRs and FPRs for sparse ceFC matrix with different choices of tuning parameter *C* = 0.0001 to *C* = 1. The colored curves correspond to the proposed thresholded estimator under different sample sizes *T*.

We further evaluate the thresholded estimator’s performance in discerning zero and nonzero entries in a sparse ceFC matrix in terms of the true positive rate (TPR; Equation 12) and the false positive rate (FPR; Equation 13), calculated as the proportion of correctly estimated nonzero entries and incorrectly estimated nonzero entries, respectively. We obtain the receiver operating characteristic curves in Figure 2 by varying the thresholding tuning parameter from *C* = 0.0001 to *C* = 1. The TPRs and FPRs, averaged over 100 scans, for *T* ∈ {50, 100, 200, 500, 1000} and *N* ∈ {50, 200} are summarized in Figure 2. We see from Figure 2 that the area under the curve increases as the sample size *T* increases, suggesting that the thresholded estimator is able to estimate the nonzero and zero entries of the true edge correlation matrix more accurately as the signal-to-noise ratio increases.

### Multiple Hypothesis Testing With Asymptotic FDR Control

We now perform statistical inference on ceFC. Specifically, we are interested in simultaneously testing the hypotheses that each element in the ceFC matrix is significantly different from zero. The literature acknowledges that not all brain regions or edges exhibit strong connections with others (Achard & Bullmore, 2007; Bullmore & Sporns, 2012; Faskowitz, Betzel, & Sporns, 2022; Faskowitz et al., 2020). Considering sparse patterns in brain networks, we construct a multiple hypothesis testing framework to systematically evaluate whether every edge-pair connection in the ceFC matrix is of strength zero. The test statistic of this inference procedure is built on the sample moment estimator, based on the asymptotic normality of the estimator. Due to the large size of the ceFC matrix, which is equivalent to testing a large number of hypotheses, we apply the Benjamini–Yekutieli procedure (Benjamini & Yekutieli, 2001) to control the FDR, providing a statistical inference procedure to systematically evaluate the strength of each edge-pair connection within the ceFC matrix. See the details in the Multiple Hypothesis Testing section.

#### Theoretical results.

Based on the multiple hypothesis testing framework, we demonstrate that the inference procedure presented in Algorithm 1 can asymptotically control the FDR, which is the expected proportion of falsely rejected null hypotheses, at a prespecified level *α*. In other words, as the number of time frames *T* increases, the FDR is asymptotically bounded above by some level *α* plus a term that converges to zero. Mathematically, it can be expressed as

$$\text{FDR}\leq \alpha +o\left(1\right).$$ (3)

Here, *o*(1) denotes a term that approaches zero, as the sample size tends to infinity. Asymptotic control of the FDR implies that for large datasets, the proportion of false positives (i.e., incorrectly identified significant edge pairs) is maintained at or below the chosen significance level *α*, ensuring the reliability of the results in large-sample scenarios. The detailed proof can be found in the Supporting Information section.

#### Simulation results for Type I error and power study.

In this section, we perform numerical studies to assess the finite-sample performance of the asymptotic normality results in the Multiple Hypothesis Testing section. Specifically, we evaluate the proposed inference framework by testing the off-diagonal entries of a sparse ceFC matrix. Our inference procedure tests the hypothesis

$$H_{0l}:\Theta_{jk,st}*=0\;vs.\;H_{1l}:\Theta_{jk,st}*\neq 0$$

for each *l*th off-diagonal entry $\Theta_{jk,st}*$ where Θ* is the true ceFC matrix. That is, we examine all possible combinations of edges to see whether each pair, denoted as *jk, st*, exhibits a strong association simultaneously. The test statistic *γ*_*jk*,*st*_ is constructed as defined in Equation 10 for each hypothesis, which is asymptotically normal as shown in Equation 8.

To evaluate the numerical performance of the inferential framework in the finite sample setting, we calculate Type I error and power as the proportion of falsely rejected and correctly rejected null hypotheses, respectively. Based on the data generation scheme described in the Simulation Settings for Inference Framework section, the centered edge connectivity matrices include both weak and moderate signals. To this end, we consider the inference for nonzero entries in two scenarios, one for moderate signals and one for weak signals that may require more samples for detection. Based on the distribution of nonzero values in the constructed ceFC matrix, we consider values within [0, 0.2] as weak signals and values within [0.2, 0.5] as moderate signals. For computational purposes, we randomly select 1,000 zero entries from the upper triangular of the edge correlation matrix for Type I error as well as 1,000 weak signals and 1,000 moderate signals for power study, respectively. This process of random selection is repeated 5,000 times. Specifically, for each of these 5,000 iterations, we calculate the Type I error based on the 1,000 zero entries and the statistical power based on the 1,000 nonzero entries (weak and moderate signals), respectively. The Type I error and power are then averaged over these 5,000 iterations and reported in Table 1. From Table 1, we can see that the Type I error is well controlled at the 0.05 significance level, and the power increases to one as the sample size increases, while we need a larger number of samples for the power of weak signals to be expected.

**Table 1.** The Type I error and power for testing the 1,000 random off-diagonal entries in edge correlation at the 0.05 significance level are averaged over 5,000 iterations. The power study is separated into 1,000 random weak signals ∈ (0, 0.2] and 1,000 random moderate signals ∈ (0.2, 0.5], respectively. Simulation results for *N* = 50 and *N* = 200 variables over a range of sample size *T* are reported.

	*N* = 50			*N* = 200		
	Type I error	Weak signal	Moderate signal	Type I error	Weak signal	Moderate signal
*T* = 300	0.048	0.237	0.858	0.048	0.163	0.755
*T* = 500	0.049	0.377	0.971	0.049	0.250	0.922
*T* = 800	0.049	0.554	0.998	0.049	0.377	0.988
*T* = 1,000	0.049	0.650	0.999	0.049	0.455	0.997
*T* = 1,500	0.050	0.813	1	0.049	0.621	1
*T* = 2,000	0.050	0.904	1	0.050	0.746	1
*T* = 3,000	0.050	0.972	1	0.050	0.894	1
*T* = 4,000	0.050	0.989	1	0.050	0.958	1
*T* = 5,000	0.050	0.994	1	0.050	0.983	1
*T* = 6,000	0.050	0.997	1	0.050	0.993	1
*T* = 7,000	0.050	0.998	1	0.050	0.997	1
*T* = 8,000	0.050	0.999	1	0.050	0.998	1
*T* = 10,000	0.050	1	1	0.050	0.999	1
*T* = 15,000	0.050	1	1	0.050	1	1

#### Simulation results for FDR control.

We further investigate the multiple hypothesis testing framework with FDR, which is computed as the proportion of falsely rejected null hypotheses among all rejections. Under the same data generation scheme described above, we choose a random subset of upper triangular entries from the ceFC matrix. Specifically, for *N* = 50 (i.e., 50 brain parcels), we select 3,000 entries, and for *N* = 200, we select 5,000 entries. These subsets of entries consist of both zero and nonzero values reflecting the sparsity of the true ceFC matrix. We summarize FDR with significance levels *α* = {0.05, 0.1, 0.2} over 5,000 datasets in Table 2. As our multiple hypothesis testing procedure is based on the procedure of Benjamini and Yekutieli (2001), our FDR results can be conservative, as illustrated in Table 2. The results in Table 2 show that the FDR is well controlled under each significance level, which is consistent with the theoretical result.

**Table 2.** The FDR for testing 3,000 random entries for *N* = 50 and 5,000 random entries for *N* = 200 are averaged over 5,000 iterations. These random subsets retain the sparsity in the corresponding edge correlation matrix. The significance level *α* sets to be {0.05, 0.1, 0.2}, and sample size *T* ranges from 400 to 700.

*N*	*α*	*T* = 400	*T* = 450	*T* = 500	*T* = 550	*T* = 600	*T* = 650	*T* = 700
*N* = 50	*α* = 0.05	0.017	0.012	0.007	0.005	0.004	0.004	0.005
	*α* = 0.1	0.020	0.015	0.011	0.009	0.009	0.009	0.011
	*α* = 0.2	0.027	0.023	0.019	0.017	0.018	0.019	0.021
*N* = 200	*α* = 0.05	0.057	0.035	0.019	0.010	0.008	0.006	0.005
	*α* = 0.1	0.060	0.035	0.022	0.015	0.012	0.009	0.009
	*α* = 0.2	0.066	0.039	0.028	0.021	0.019	0.016	0.017

### Identifying Overlapping Communities

A key feature of the edge-centric framework is that it allows direct and natural identification of overlapping communities. Substantial evidence supports the existence of overlapping structures in brain networks (Beckmann et al., 2005; Palla et al., 2005; van den Heuvel & Sporns, 2013; Yeo et al., 2014), partly motivated by responses to the extensive body of literature studying nonoverlapping communities (Power et al., 2011; Yeo et al., 2011). In this section, we demonstrate how the proposed estimators for ceFC can be used to identify overlapping communities and provide deeper insights into brain network organization (Chumin et al., 2022; Faskowitz et al., 2020), which can be potentially developed as biomarkers of various disorders (Dai et al., 2023; Sun et al., 2023; Yang et al., 2023; Zamani Esfahlani et al., 2022).

We apply our proposed methods to the MSC data (Gordon et al., 2017) with *N* = 200 brain regions of interest. We then perform spectral clustering on the estimated ceFC matrix to identify overlapping communities of the brain regions. Specifically, we estimate the ceFC for each subject using (a) the sample moment estimator in Equation 6; (b) the FDR-controlled estimator, obtained by testing whether each entry of the computed sample moment estimator is equal to zero with the FDR controlled at a level of 0.1, and zeroing insignificant entries; and (c) the thresholded estimator in Equation 11.

The resulting FDR-controlled estimators exhibit slightly varying degrees of sparsity, with levels ranging from 0.6 to 0.8 across all subjects. The thresholded estimator (Equation 11) involves selecting a tuning parameter *τ*—we select *τ* such that the sparsity level of the thresholded estimator aligns with that of the FDR-controlled estimator for each subject. Finally, we compute the average for each of the three estimators across 10 subjects.

After obtaining edge communities by spectral clustering, we project them back to the brain region level following the procedure in Faskowitz et al. (2020) to obtain overlapping communities for the brain regions. See the Clustering Procedure section for details.

**Figure 3.**
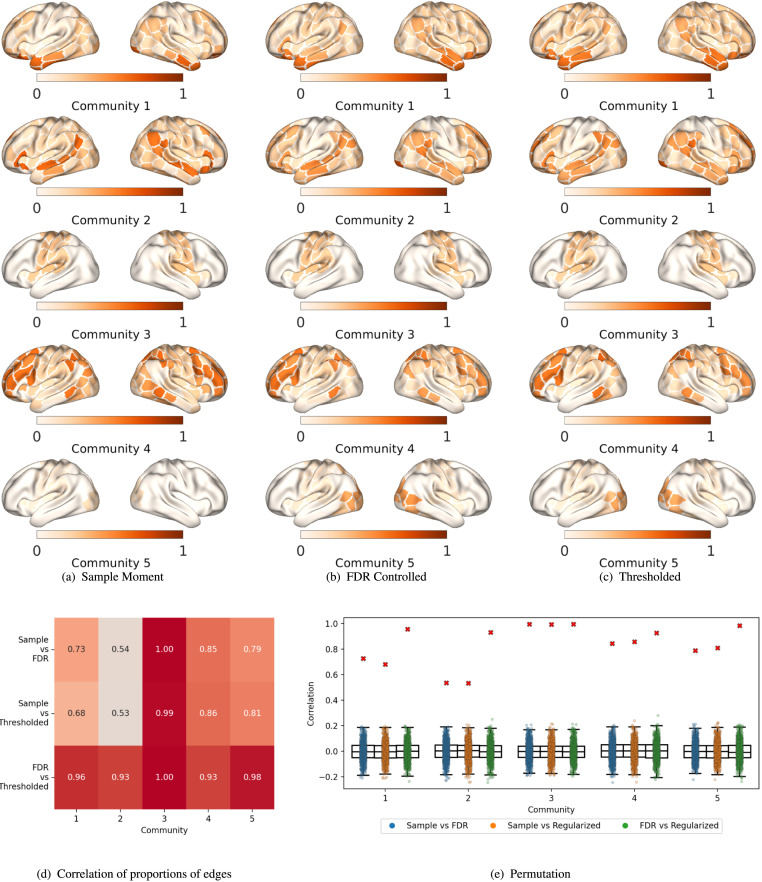
For all edges connected to each brain region, we compute the proportions of edges within each community. Plots A–C correspond to results based on the average sample moment estimator, the average FDR-controlled estimator with FDR controlled at level 0.1, and the average thresholded estimator, respectively. Plot D shows the correlation of proportions of edges within each community in plots A–C between each pair of methods. Plot E shows the correlation of proportions of edges within each community between each pair of methods under random permutations (1,000 iterations). The observed correlations from the original results are marked as red “X”s.

For visualization purposes with sufficiently distinct yet not overly scattered clusters, we present the results for five communities in Figures 3A, 3B, and 3C, representing results based on the sample moment estimator, the FDR-controlled estimator, and the thresholded estimator, respectively.

These results in general show that the three different estimators reveal similar community structures (ANOVA: *F*(2, 2997) = 0.1, *p* = 0.9. Each observation represents the proportion of edges assigned to a specific community for a given brain region as shown in Figures 3A, 3B, and 3C, with 200 brain regions × 5 communities per estimator). Specifically, the sample moment estimator, as shown in the Figure 3A, predominantly identifies communities 1, 2, and 4, with the average proportions across all brain parcels as 25.7%, 31.5%, and 29.3%, respectively. In contrast, both FDR-controlled (Figure 3B) and thresholded estimators (Figure 3C) additionally group the brain regions into communities 1, 2, 4, and 5 with similar proportions. Specifically, the FDR-controlled estimator shows average proportions for these four communities as 29.8%, 26.9%, 23%, and 11.5%, respectively, and the thresholded estimator returns similar outcomes as 31.8%, 28.3%, 20.8%, and 10.7%. The similarity across the three methods can be further quantified through the correlation of proportions of edges within each community between each pair of methods. As shown in Figure 3D, all three methods present highly correlated community structures, with more similar results between the FDR-controlled and thresholded estimators, demonstrating that the identified community structures are robust to the specific statistical pipeline applied to the ceFC results. We further examine the significance of these correlations against a null model using a spin permutation test. Specifically, we randomly shuffle the 200 brain parcels 1,000 times while preserving their spatial autocorrelation structure. The corresponding correlation is then computed for each shuffle to generate a null distribution. The results are summarized in Figure 3E, where the observed correlations for all comparisons were significantly higher than the values generated from the permuted data (*p* < 0.001 from 1,000 data points or shuffles), suggesting these similarities are significantly higher than what is expected given the spatial autocorrelation of the fMRI data when the indices are shuffled.

**Figure 4.**
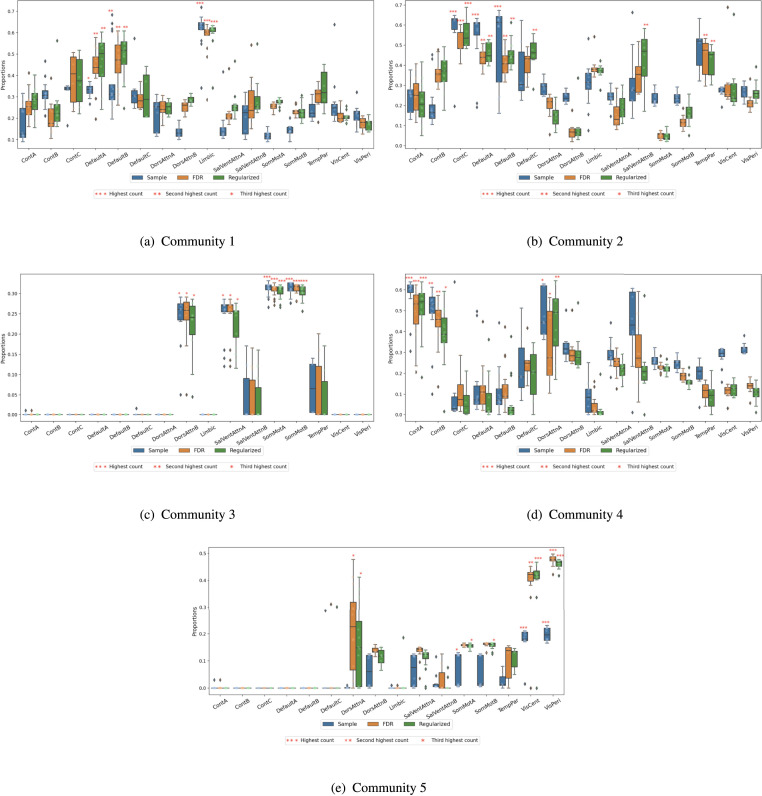
Proportions of edges within each community and associated brain systems as depicted in Figure 3. The plot highlights the top three brain systems that demonstrate a significantly greater proportion of edges in pairwise comparisons with other systems. * * * denotes the system with the highest count of significant outcomes, * * represents the second highest, and * signifies the third highest.

The projection patterns indicate varying involvement of brain systems across different communities. Specifically, community 1 is dominated by default and limbic networks; community 2 is primarily influenced by default, control, and temporoparietal networks; community 3 is mostly composed of somatomotor and attention networks; community 4 mainly comprises control and attention networks; and community 5 is primarily associated with attention, somatomotor, and visual networks. Note, however, that nodal memberships in each community are graded. For instance, community 2 from the sample moment estimator (Figure 3A) is, indeed, dominated by default mode regions—that is, those regions are most strongly affiliated with this community—but even somatomotor regions exhibit weak, but nonnegligible, affiliation. To systematically assess the significant proportion differences among these brain systems, we first identified the presence of any disparities among brain systems within each community and then applied Tukey’s HSD test for multiple comparisons between each pair of brain systems, utilizing adjusted *p* values at a significance level of 0.05. For each system, we compiled the number of outcomes where a system showed significantly greater proportions than others and summarized these findings in Supporting Information Figure S1, reporting results for each community and method. The top three systems with the highest counts of significant outcomes are highlighted in Figure 4. Here, * * * indicates the system with the highest number of significant outcomes, * * denotes the second highest, and * signifies the third highest, reflecting the levels of significance of the proportions within each system.

To evaluate the overlapping structures across communities, we compute the normalized entropy using the proportions across the five communities within each brain system. This metric measures the uncertainty of community delineations: Values closer to 1 indicate greater overlap across communities, while values closer to 0 suggest a more concentrated distribution within a single community. The results, displayed in Supporting Information Figure S2, show higher entropy values in the attention and somatomotor networks. On the surface, these findings of substantial functional overlap in these specific networks are consistent with findings from a previous study (Faskowitz et al., 2020). However, our ceFC framework provides a more robust interpretation of this result by addressing a critical source of bias in the eFC definition that could impact reproducibility. Mathematically, eFC is sensitive to the mean of edge time series (i.e., static functional connectivity), introducing a sample-specific bias. In contrast, our proposed ceFC is, by definition, a centered covariation that explicitly removes such bias.

The similarity of our results to those from eFC, despite the removal of the confounding bias, suggests that while the original eFC findings were directionally correct, they possess an inherent vulnerability that their reproducibility in future studies would depend on new datasets sharing the same static connectivity biases. Our ceFC results, therefore, are not a simple replication. Instead, they serve as a crucial validation, isolating the true, reproducible phenomenon from a potentially biased observation.

With this refined understanding, we further compare the normalized entropy between each pair of brain systems and record the number of significantly higher entropy outcomes for each system, as shown in Supporting Information Figure S3. This analysis further indicates that the aforementioned brain systems, that is, attentional and somatomotor networks, exhibit significantly greater overlapping structures.

While previous results show broadly similar patterns across the three estimators, there are certain differences among them. In contrast to the FDR-controlled and thresholded estimators, the sample moment estimator reveals subtle variances in community structures, particularly among systems associated with functional tasks. It is not surprising to observe similar results between the FDR-controlled and thresholded estimators, as the tuning parameter *τ* in the thresholded estimator is chosen to align its sparsity with that of the FDR-controlled estimator. Specifically, Figure 4 indicates that the somatomotor networks are more active in community 1 by the FDR-controlled and thresholded estimators but in community 2 by the sample moment estimator. Similarly, the visual systems are more prominent in community 5 by the FDR-controlled and thresholded estimators, but are relatively more effective in community 4 by the sample moment estimator. In contrast, the somatomotor and temporoparietal systems exhibit higher activity in community 5 by FDR-controlled and thresholded estimators, but tend to be more effective in community 4 by the sample moment estimator. Previous studies have highlighted the significant engagement of the somatomotor and visual systems in respective motor and visual functions (Biswal, Zerrin Yetkin, Haughton, & Hyde, 1995; Chenji et al., 2016; Yang, Deng, Xing, Xia, & Li, 2015). These distinctions underscore the value of exploring system behavior related to functional tasks within a resting-state context.

**Figure 5.**
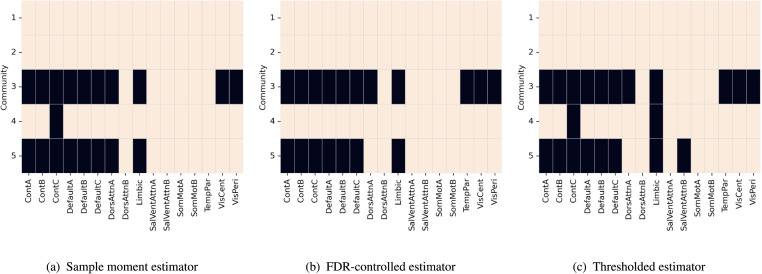
Brain systems with significant proportions from each average estimator under the significance level of 0.05. The black box represents the insignificant result, and the yellow box represents the significant result.

We now perform a formal statistical hypothesis test to assess whether the average proportion within each brain system significantly exceeds zero. Specifically, for each system *s*, we compute its average proportion *μ_cs_* within each community *c* and test the following hypothesis:

$$H_{0}:\mu_{cs}=0\;vs.\;H_{1}:\mu_{cs}>0$$ (4)

with a significance level of 0.05. While the null model may fail to preserve the spatial structure of the original data (Váša & Mišić, 2022), our objective is to evaluate the performance of these estimators statistically. Therefore, we proceed with the same testing procedure for the proportions from each subject-specific estimator and the average estimator, respectively. For visualization purposes, the test results from the average estimators are summarized in Figure 5. The results in Figure 5 indicate general consensus among the three estimators regarding the systems with significant proportions within communities, although subtle disparities are observed in communities 3, 4, and 5.

### Stability of the Proposed Estimators

In this section, we conduct an extensive sensitivity analysis to evaluate the stability of the three estimators across different individuals. See the Sensitivity Analysis section for details. In general, we assess the stability of the three estimators by juxtaposing the previously mentioned test outcomes from individual participants with those derived from their respective average estimators and then compute the FPR and the false negative rate (FNR) as shown in Equation 14, defined as the proportion of communities or systems incorrectly identified as significant and the proportion of communities or systems incorrectly identified as insignificant across all communities, respectively.

**Figure 6.**
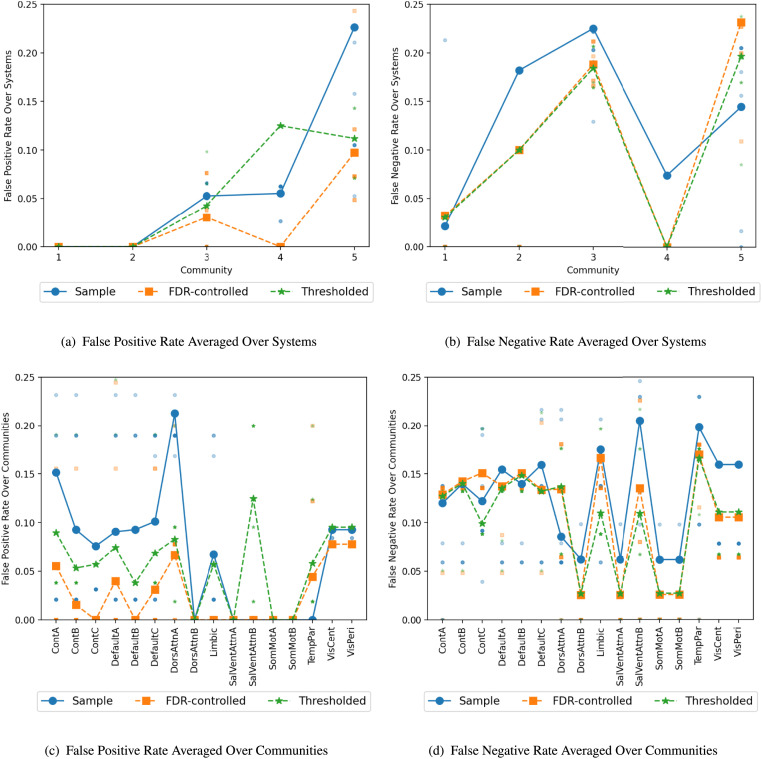
The FPRs and FNRs are averaged across 16 systems (first row) or five communities (second row). The solid points on line graphs represent the average results over subjects, and each small transparent point corresponds to each subject’s result.

The results averaged over the 10 subjects are reported in Table 3. It can be seen from Table 3 that both FDR-controlled and thresholded estimators achieve lower FPRs and FNRs compared with the results from the sample moment estimator. Specifically, the FPRs and FNRs are reduced by 61% and 15% in the FDR-controlled estimator and reduced by 16% and 21% in the thresholded estimator, respectively. Additionally, we compute the average FPRs and average FNRs for all three methods at the community and brain system levels, respectively. The results are displayed in Figure 6. We see from Figure 6 that the sample moment estimator generally demonstrates comparable or higher FPRs and FNRs across the majority of communities and brain systems. The FDR-controlled and thresholded estimators exhibit similar FNRs, while the thresholded estimator returns slightly higher FPRs in certain communities and brain systems. Our results suggest that the proposed FDR-controlled and thresholded estimators, which disregard the noise and weak signals in edge connectivity, are likely to yield more stable outcomes than the sample moment estimator.

**Table 3.** FPR and FNR are averaged across 16 brain systems and five communities over 10 subject data for each estimator.

Method	FPR	FNR
Sample moment	0.067	0.129
FDR-controlled	0.026	0.110
Thresholded	0.056	0.102

## DISCUSSION

In this study, we focus on the unbiased covariation between pairs of edges termed ceFC, where we remove the effect of the mean of edge time series and provide a new perspective on relationships among edges. We introduce the sample moment estimator under a fixed-*N* regime and establish an inferential framework for multiple hypothesis testing with a controlled FDR for evaluating the covariation among edges. We further extend our study to a high-dimensional setting with a thresholded estimator. Applications of the proposed methods on fMRI data illustrate how the analysis of edge-centric connections can help identify more stable overlapping brain community structures and provide a new perspective on brain organization.

One objective of this analysis is to establish the theoretical foundations for estimation performance, specifically focusing on the statistical rate of convergence to the true parameter, where we relax the assumption under sub-Gaussian variables. We demonstrate that the proposed estimators can converge to the true ceFC matrix at a rate that primarily depends on the number of variables or brain parcels *N*, where the convergence rate of the thresholded estimator is further influenced by the number of nonzero entries, *m*, in a sparse ceFC matrix. Therefore, even with a limited number of time frames, *T*, the thresholded estimator can achieve a reliable estimation of a sparse ceFC matrix, with estimation error converging to zero.

Our application of the ceFC framework to the MSC dataset revealed a complex, overlapping community structure. In our findings, attentional and somatomotor networks predominantly occupy communities 3 and 5, with the attentional network further dominating community 4; the default network dominates communities 1 and 2, and the control network is active in communities 2 and 4. A key finding was the prominent overlap between the attentional and somatomotor networks, a result that, on the surface, aligns with previous edge-centric analyses (Faskowitz et al., 2020). However, a crucial contribution of our work lies in the disambiguation of this finding. The original eFC framework is sensitive to the mean of edge time series (i.e., static functional connectivity), introducing a sample-specific bias that can conflate the effects of static connectivity strength with those of dynamic cofluctuations. It was therefore unclear whether previously observed network overlaps were driven by persistently strong connections or by synchronized dynamic adjustments.

Our ceFC framework resolves this ambiguity. By providing an unbiased estimate of edge covariation, the fact that the overlapping community structure persists in our analysis is therefore not a simple replication but a validation. It provides evidence that this network architecture is a phenomenon rooted in higher-order coordination without the incorporation of static connectivity bias. This distinction is vital for scientific reproducibility. The findings from our ceFC framework are more likely to be stable across new datasets, even those with different static connectivity profiles. This stability arises because static connectivity itself is a primary source of sample-specific variability, where the intersubject standard deviations range from 0.02 to 0.37 in MSC data. By isolating such components, ceFC captures a more generalizable and reproducible representation of higher-order relationships within the network architecture.

This principle of isolating a robust, reproducible signal is further supported by our sensitivity analysis. We found that both the FDR-controlled and thresholded estimators produce more stable overlapping community structures compared with the basic sample moment estimator. This enhanced stability stems from their ability to effectively filter out noise and weak signals, thereby capturing the strong, underlying dynamic organization that our ceFC framework is designed to isolate.

### Limitations and Future Directions

While our thresholded estimator provides statistical advantages in high-dimensional settings, it shares with other sparsity-based methods the potential removal of biologically meaningful weak connections. Previous studies have highlighted the functional importance of weak ties in brain networks (Gallos, Makse, & Sigman, 2012; Park & Friston, 2013; Varley, Pope, Faskowitz, & Sporns, 2023). However, with limited sample sizes typical in fMRI studies, it becomes statistically challenging to reliably distinguish genuinely weak biological signals from random noise across the *O*(*N*^4^) potential edge–edge connections. Our thresholding framework prioritizes the identification of robust, reproducible higher-order interactions while controlling false discoveries, though we recognize that some subtle yet important connections might be excluded. Future studies with larger sample sizes or multisite datasets may enable more powerful detection of subtle yet meaningful higher-order interactions.

Moreover, although ceFC effectively captures the higher-order connectivity in brain networks, it is limited in revealing the linear connections between edges. Considering the intricate and nontrivial structures inherent in brain networks and nervous systems (Lahaye, Poline, Flandin, Dodel, & Garnero, 2003; Roberts & Robinson, 2012; Spiegler, Knösche, Schwab, Haueisen, & Atay, 2011; Stephan et al., 2008), such a linear measurement may not fully capture the complex nonlinear patterns that could enhance sensitivity analysis across groups of people (Kinsey et al., 2024; Motlaghian et al., 2022). Therefore, it is meaningful to explore additional metrics that measure nonlinear relationships among edges, such as distance correlation (Székely, Rizzo, & Bakirov, 2007) for dependency measurement, providing a more comprehensive understanding of brain organizations.

The application of higher-order relationships in the analysis of brain networks provides new insights into functional brain organizations. Our study so far focuses on analyzing connectivity patterns within each subject without extending the analysis to include the comparison across a group of subjects, a limitation that does not account for potentially shared patterns or intersubject variability. Looking forward, one promising direction includes the multisubject analysis, exploring both the group-wise pattern among multiple subjects and the individual-specific characteristics of each subject. Understanding group-wise patterns could assist in disease diagnosis via the identification of biomarkers, and examining the individual-specific characteristics could provide insights into personalized medicine through personal information, extending current edge-based studies of group-wise comparison across healthy people and patients (Dai et al., 2023; Sun et al., 2023; Yang et al., 2023; Zamani Esfahlani et al., 2022) and individual differences (Ville et al., 2021).

## METHODS

### MSC Dataset

The MSC data (Gordon et al., 2017) consists of fMRI images of 10 healthy adults (5 female participants, age 24–34 years). Informed consent was obtained from all participants, and the study received approval from the Human Studies Committee and Institutional Review Board at Washington University School of Medicine. Each participant underwent ten 30-min scan sessions with eyes open on separate days, with each scan starting from midnight. All images, including four T1-weighted and four T2-weighted images, were collected by a Siemens TRIO 3T MRI scanner. For a detailed description of data acquisition, please see Gordon et al. (2017).

Hand-edited cortical surfaces were generated based on average T1 images from high-resolution structural MRI measurements using Freesurfer (Dale, Fischl, & Sereno, 1999) and were registered into fs LR 32k surface space (Glasser et al., 2013). In addition, the fs LR 32k surface space could be transformed into Talairach volumetric space by a separate computation on the average native T1-to-Talairach transformation (Talairach & Tournoux, 1988).

These fMRI measurements were preprocessed with slice-time correction, motion correction, intensity normalization, atlas transformation with registration to T2 image, and distortion correction (Gordon et al., 2017). Subsequently, the preprocessed fMRI data underwent demeaning and detrending processes, nuisance regression, a high-motion frame dropping with framewise displacement over 0.2 mm, and bandpass filtering between 0.009 Hz and 0.08 Hz (Power et al., 2014). These functional measurements were further transposed to the cortical surface with a two-dimensional geodesic smoothing.

The preprocessed resting-state fMRI measurements were aggregated within each subject, resulting in 4,000–8,000 time frames for 10 subjects, that is, *T* ranges from 4,000 to 8,000. These BOLD signals were mapped onto the Schaefer200 parcellation (Schaefer et al., 2018), defining 200 brain regions of interest, or equivalently *N* = 200. These brain regions can be further grouped into canonical functional networks based on the system-level structure proposed by Yeo et al. (2011).

### ceFC

Functional connectivity measures the magnitude of dependency between a pair of brain voxels or parcels. One common measurement is the Pearson correlation coefficient using their BOLD signal time series. Let ***x****_j_* = [*x*_1*j*_, …, *x*_*Tj*_] denote the time series recorded from parcel *j*, where *T* is the number of time frames and *N* is the number of parcels. For each parcel *j*, we compute the time-averaged mean $\mu_{j}=\frac{1}{T}\sum_{i}x_{ij}$ and standard deviation $\sigma_{j}=\sqrt{\frac{1}{T-1}\sum_{i}{\left(x_{ij}-\mu_{j}\right)}^{2}}$, such that we can obtain the *z*-scored time series vector $z_{j}=\frac{x_{j}-\mu_{j}}{\sigma_{j}}$. For any pair of *z*-scored time series vectors ***z****_j_*, ***z****_k_*, the correlation between parcels *j* and *k* can be computed as $r_{jk}=\frac{1}{T-1}\sum_{i}\left[z_{ij}\cdot z_{ik}\right]$. By repeating this computation for all pairs of parcels, we obtain the node-by-node correlation matrix ***R*** = [*r*_*jk*_] of size *N* × *N*, which is the estimate of node-centric functional connectivity (nFC). Here, we treat brain parcels as random variables and use uppercase letters to denote them. Specifically, let *X*_1_, …, *X*_*N*_ be the random variables for the *N* brain parcels, and let ***X*** = (*X*_1_, …, *X*_*N*_)*^T^* be an *N*-dimensional random vector with mean zero and an *N* × *N* covariance matrix **Σ** = [*σ_jk_*] ∈ ℝ^*N* × *N*^. The above computation for the correlation can be written as $r_{jk}=\frac{E\left[X_{j}X_{k}\right]}{\sigma_{j}\sigma_{k}}$, where *σ_j_, σ_k_* are the standard deviations of *X*_*j*_, *X*_*k*_, respectively.

To model higher-order dependencies among brain parcels, we define ceFC among parcels {*j*, *k*} and {*s*, *t*} as

$$\Theta_{jk,st}=E\left[X_{j}X_{k}X_{s}X_{t}\right]-E\left[X_{j}X_{k}\right]∙E\left[X_{s}X_{t}\right].$$ (5)

Here, Θ_*jk*,*st*_ measures linear dependencies between two pairs of parcels {*j*, *k*} and {*s*, *t*}. If we consider the *z*-scored BOLD signals *Z*_1_, …, *Z*_*N*_, Θ_*jk*,*st*_ can be viewed as the connection between edge *jk* and edge *st* by taking the product of centered edge time series *Z*_j_ *Z_k_* and *Z*_s_ *Z_t_*. Moreover, if we standardize the edge time series *Z*_j_ *Z_k_* and *Z*_s_ *Z_t_* and then take the product, the resultant ceFC will be the correlation between edge *jk* and edge *st* and thus scaled between  [−1, 1]. Considering all combinations of parcel pairs for entries in ceFC, Θ will be a $\frac{N\left(N-1\right)}{2}\times \frac{N\left(N-1\right)}{2}$ matrix. In the following sections, we will propose two types of estimators for ceFC under low- and high-dimensional settings, respectively.

### Null Hypothesis

As the ceFC is constructed based on BOLD signal series *X*_1_, …, *X*_*N*_, we impose some condition or null hypothesis on the distribution of vector ***X***. Specifically, we assume the random vector ***X*** ∈ ℝ*^N^* is *sub-Gaussian* with mean zero and covariance matrix $\Sigma =E\left[{XX}^{T}\right]\in {\mathbb{R}}^{N\times N}$ that is positive definite. Consequently, for each *X*_*j*_ and *p* ≥ 1/4, we have $E\left[{\left|X_{j}\right|}^{4p}\right]\leq K^{4p}{\left(4p\right)}^{2p}$ for some constant *K* > 0.

### Sample Estimation of ceFC

To estimate ceFC Θ_*jk*,*st*_ defined in Equation 5, we consider the situation where we have a sufficient sample size *T* compared with the number of variables *N*. In other words, the number of time frames of BOLD signals is large enough compared with the number of brain parcels. Let ***X***_1_, …, ***X****_T_* be *T* independently and identically distributed copies of ***X***, which represent the BOLD time series of whole brain parcels. A natural estimator of Θ_*jk*,*st*_ is constructed by the sample mean of each expectation term in Equation 5, which is the following sample moment-based estimator:

$$\begin{array}{ll} {\hat{\Theta }}_{jk,st}= & \frac{1}{T}\sum_{i=1}^{T}X_{ij}X_{ik}X_{\text{is}}X_{it}\\ & -\frac{1}{T}\sum_{i=1}^{T}X_{ij}X_{ik}\cdot \frac{1}{T}\sum_{i=1}^{T}X_{\text{is}}X_{it}. \end{array}$$ (6)

If we repeat this computation for all combinations of parcel pairs, we will obtain a $\frac{N\left(N-1\right)}{2}\times \frac{N\left(N-1\right)}{2}$ matrix $\hat{\Theta }$, a sample estimate of Θ.

### Connection With nFC

As the definition for ceFC (Equation 5) involves connectivity between nodes, it is of interest to study the connection of the ceFC matrix with the nFC matrix. Indeed, Magnus and Neudecker (1979) have shown the mathematical relationship between a covariance matrix and a ceFC-like matrix under a Gaussian distribution based on the communication matrix. By definition, a communication matrix ***K***_*m*,*n*_ is a square *mn* × *mn* matrix consisting of *mn* submatrices and the (*i*th, *j*th) submatrix ***K***_*i*,*j*_ contains 1 in its (*i*th, *j*th) entry and 0 otherwise. When *m* = *n*, we can simplify the notation ***K***_*n*,*n*_ to ***K****_n_*. Specifically, Magnus and Neudecker (1979) state that for a Gaussian random vector ***X*** ∈ ℝ*^N^* with mean $\tilde{\mu }$ and covariance $\tilde{\Sigma }$,

$$\text{Var}\left(X⊗X\right)=\left(I+K_{p}\right)\left(\tilde{\Sigma }⊗\tilde{\Sigma }+\tilde{\Sigma }⊗\tilde{\mu }{\tilde{\mu }}^{\prime }+\tilde{\mu }{\tilde{\mu }}^{\prime }⊗\tilde{\Sigma }\right),$$

where ⊗ denotes the Kronecker product. Here, Var(***X*** ⊗ ***X***) can be viewed as an extension of ceFC, which includes the covariance between the node pairs {*X*_*j*_, *X*_*k*_} and {*X*_*s*_, *X*_*t*_} and also the case when *j* = *k* and/or *s* = *t*. Our study ignores the second case in **Θ**, as there is no edge connecting the node *X*_*j*_ itself. Based on the result in Magnus and Neudecker (1979), when the random vector ***X*** = (*X*_1_, …, *X*_*N*_)^T^ is Gaussian with mean **0** and an *N* × *N* covariance matrix **Σ** = [*σ_jk_*] ∈ ℝ^*N*×*N*^, we can rewrite

$$\Theta =\left(I+K_{N,N-1}\right)\left(\Sigma ⊗\Sigma_{-j,-k}\right)\in {\mathbb{R}}^{N\left(N-1\right)\times N\left(N-1\right)}$$ (7)

for *j*, *k* ∈ {1, …, *N*}. There are two modifications from Magnus and Neudecker’s (1979) result. First, ***K***_*N*,*N*−1_ is a variation of the communication matrix ***K****_N_*, which contains *N* × (*N* − 1) submatrices each with a dimension of (*N* − 1) × (*N* − 1). In particular, the (*j*th, *k*th) submatrix contains 1 in its (*j* − 1th, *k*th) entry when *j* > *k* and (*j*th, (*k* − 1)th) entry when *j* < *k*, and 0 elsewhere. Second, the matrix **Σ**_−*j*,−*k*_ removes *σ_jk_* from **Σ**. Specifically, **Σ** ⊗ **Σ**_−*j*,−*k*_ = [*σ_jk_***Σ**_−*j*,−*k*_]. Such modifications are mainly based on the removal of the term *σ_jj_* for *j* ∈ {1, …, *N*}, which does not retain practical meaning in some application fields.

When considering the special case of *z*-scored node time series, Equation 7 provides the mathematical relationship between ceFC and nFC, indicating that the ceFC matrix can be built from nFC. This may not be surprising as the construction of ceFC matrix depends on the edge time series, which in turn is obtained based on the node time series.

### Comparison With eFC

The concept of ceFC is closely related to eFC as proposed by Faskowitz et al. (2020). Both ceFC and eFC can quantify the connection between pairs of edges but from different perspectives. This distinction has profound implications for the interpretation and reproducibility of findings in eFC.

Specifically, eFC calculates the strength of edge connections based on the inner product of two edge time series, while ceFC is evaluated using centered edge time series. Mathematically, let *E*_*jk*_ denote the edge time series between parcels *j* and *k*, which can be obtained by taking the element-wise product of *X*_*j*_ and *X*_*k*_ as (*X*_1*j*_*X*_1*k*_, …, *X*_Tj_ *X_Tk_*). The root-mean-square of the edge time series can be defined as $∥E_{jk}∥=\sqrt{E_{jk}^{T}E_{jk}/T}$. The standard deviation of *E*_*jk*_ is $sd\left(E_{jk}\right)=\sqrt{{\left(E_{jk}-\sigma_{jk}\right)}^{T}\left(E_{jk}-\sigma_{jk}\right)/\left(T-1\right)}$, where *σ_jk_* is the mean of the edge time series *E*_*jk*_, or the covariance between parcels *j* and *k* with zero-mean node time series. By definition, eFC between edges *jk* and *st* can be computed as

$${eFC}_{jk,st}=\frac{E_{jk}^{T}E_{st}/T}{∥E_{jk}∥∥E_{st}∥}.$$

Similarly, we can obtain ceFC between edges *jk* and *st* under the scale between −1 and 1 by

$${\text{ceFC}}_{jk,st}=\frac{E_{jk}^{T}E_{st}/T-\sigma_{jk}\sigma_{st}}{sd\left(E_{jk}\right)sd\left(E_{st}\right)}.$$

The critical distinction lies in the centering step. The presence of the product of means, *σ_jk_σ_st_*, in the eFC formulation introduces a systematic bias that conflates the covariation between edge time series with the magnitudes of their individual means. Consequently, two edge pairs might exhibit high eFC not because their fluctuations are synchronized, but because both pairs have strong average connections. This confound makes eFC results dependent on sample-specific mean connectivity levels, threatening the reproducibility and generalization of findings across studies with different populations or acquisition protocols.

To empirically illustrate the impact of this centering step, we directly compare the value distributions of the eFC and ceFC matrices using the sample moment estimator from simulation with *N* = 200 brain parcels. The details can be found in the Simulation Settings for Estimation Evaluation section. We visualize entry values in eFC, ceFC, and their difference in Supporting Information Figure S4. In general, eFC and ceFC exhibit similar patterns in that both their mean values (eFC: 0.0019, ceFC: 0.0016) and standard deviations (eFC: 0.073, ceFC: 0.065) are close, and their difference ranges from −0.24 to 0.33. Due to the large sample size and small standard deviations, a standard *t* test is overly sensitive to negligible differences and is not informative. Consequently, we instead compare the element-wise estimation bias from the upper triangular entries of eFC and ceFC, both of which are symmetric matrices with unit diagonals. To quantify the extent of overestimation relative to the ground truth Θ, we compute the overestimation ratio for each off-diagonal entry in the estimators where the estimator's absolute value exceeds the corresponding true absolute value. Specifically, for such entries, we define the overestimation ratio as $\left(|{\hat{\Theta }}_{jk,st}|-|\Theta_{jk,st}|\right)/|\Theta_{jk,st}|\times 100\%$. This ratio captures the relative magnitude of overestimation with respect to the true value. As illustrated in Supporting Information Figure S4D, the distribution of these ratios, plotted on a logarithmic scale, reveals that the eFC estimator exhibits systematically higher overestimation compared with ceFC, underscoring the biased estimation in eFC. This analysis provides empirical evidence that the centering step, that is, *σ_jk_σ_st_* term, is nontrivial and essential for accuracy. The consistently lower overestimation ratios of ceFC validate our theoretical concern that omitting this term introduces bias and leads to a less reliable estimation.

**Figure 7.**
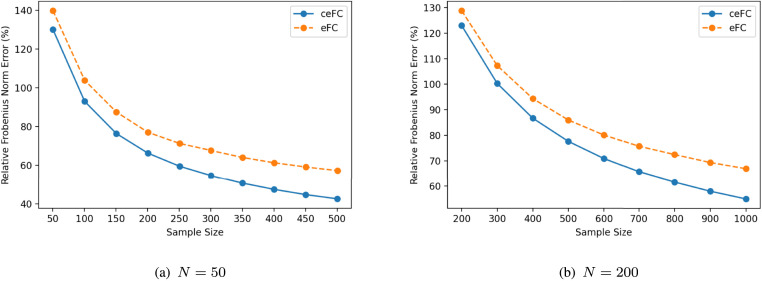
Relative Frobenius norm estimation error for a nonsparse ceFC matrix. The colored lines correspond to the proposed sample moment estimation for ceFC (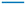) and eFC (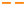).

When the parameter of interest is the unbiased covariation between edge pairs, ceFC provides superior estimation accuracy, as demonstrated by the estimation error under the Frobenius norm. Under the same simulation setting described in the Simulation Settings for Estimation Evaluation section, both eFC and ceFC based on sample moment estimators show decreasing estimation errors with increasing sample size, but ceFC consistently achieves smaller errors that converge to zero at a faster rate as illustrated in Figures 7A and 7B.

Beyond estimation accuracy, it is crucial to assess a method’s empirical robustness. To formally compare the robustness of eFC and ceFC, we design a split-half reliability simulation with details described in the Simulation Settings for Split-Half Reliability section. The results are summarized in Supporting Information Figure S5, which displays the overall distributions of split-half reliability scores across all edge pairs for *N* = 50 and *N* = 200 parcels. Overall, ceFC and eFC demonstrate comparable reliability distributions. However, closer inspection reveals that ceFC shows a marginally higher count of edge pairs with positive split-half correlations, whereas eFC yields relatively more edge pairs with negative correlations. Although these distributional differences are visually indistinguishable by the large count scale in the plot, given the total number of edges considered, even minor proportional shifts can correspond to a substantial number of connections, suggesting potentially methodological distinctions in reliability behavior.

Therefore, even if eFC and ceFC were to yield visually similar community structures in a given dataset, the communities identified by eFC are built upon a biased foundation, where the similar patterns could be dependent on the specific datasets, while those from ceFC reflect a more reproducible signature of higher-order brain dynamics. In the special scenario where parcel pairs *j, k* and *s, t* are both uncorrelated (*σ_jk_* = 0 and *σ_st_* = 0) with sufficient time frames, the centering step becomes unnecessary and ∣*E*_*jk*_∣ equals *sd*(*E*_*jk*_), making *eFC*_*jk, st*_ equivalent to *ceFC*_*jk,st*_ after standardization. However, this represents an edge case rather than the typical scenario in functional neuroimaging.

### Multiple Hypothesis Testing

We now conduct multiple hypothesis testing on ceFC entries and check whether each of them is zero or not. Formally, our goal is to test each *l*th edge pair of {*jk*, *st*} in **Θ** simultaneously:

$$H_{0l}:\Theta_{jk,st}=0\;vs.\;H_{1l}:\Theta_{jk,st}\neq 0,\;l=1,\ldots ,M.$$

Given that the ceFC matrix **Θ** is symmetric with nonzero diagonals, it is sufficient to examine the set of *M* upper triangular entries of **Θ** for the *M* hypotheses with *M* = $\frac{N\left(N-1\right)\;\left[N\left(N-1\right)-2\right]}{8}$.

It remains to construct a test statistic for the multiple hypotheses based on the proposed sample moment estimator ${\hat{\Theta }}_{jk,st}$. By an application of the multivariate central limit theorem and multivariate delta method, it can be shown that

$$\sqrt{T}\left[{\hat{\Theta }}_{jk,st}-\Theta_{jk,st}\right]\overset{d}{\to }N\left(0,\Lambda_{jk,st}\right),$$ (8)

where

Λjk,st=VarXjXkXsXt+σst2VarXjXk+σjk2VarXsXt−2σstCovXjXk,XjXkXsXt−2σjkCovXsXt,XjXkXsXt+2σjkσstCovXjXk,XsXt. (9)

Under the null hypothesis, we define the test statistic as

$$\gamma_{jk,st}=\frac{{\hat{\Theta }}_{jk,st}}{\sqrt{{\hat{\Lambda }}_{jk,st}/T}},$$ (10)

where ${\hat{\Lambda }}_{jk,st}$ is the sample estimate of Λ_*jk*,*st*_. For ease of notation, we use the subscript *c* to denote the centered product of certain BOLD signal series. That is,

XijXikc=XijXik−T−1∑i=1TXijXik,XisXitc=XisXit−T−1∑i=1TXisXit,XijXikXisXitc=XijXikXisXit−T−1∑i=1TXijXikXisXit.

With these centered products, we can write the sample estimate ${\hat{\Lambda }}_{jk,st}$ in terms of the sample estimate for each variance or covariance part in Equation 9 as

$$\begin{array}{l} \frac{1}{T-1}\sum_{i=1}^{T}{\left(X_{ij}X_{ik}X_{\text{is}}X_{it}\right)}_{c}^{2}\\ +{\left(\frac{1}{T}\sum_{i=1}^{T}X_{\text{is}}X_{it}\right)}^{2}\cdot \frac{1}{T-1}\sum_{i=1}^{T}{\left(X_{ij}X_{ik}\right)}_{c}^{2}\\ +{\left(\frac{1}{T}\sum_{i=1}^{T}X_{ij}X_{ik}\right)}^{2}\cdot \frac{1}{T-1}\sum_{i=1}^{T}{\left(X_{\text{is}}X_{it}\right)}_{c}^{2}\\ -2\left(\frac{1}{T}\sum_{i=1}^{T}X_{\text{is}}X_{it}\right)\cdot \frac{1}{T-1}\sum_{i=1}^{T}{\left(X_{ij}X_{ik}\right)}_{c}{\left(X_{ij}X_{ik}X_{\text{is}}X_{it}\right)}_{c}\\ -\;2\left(\frac{1}{T}\sum_{i=1}^{T}X_{ij}X_{ik}\right)\cdot \frac{1}{T-1}\sum_{i=1}^{T}{\left(X_{\text{is}}X_{it}\right)}_{c}{\left(X_{ij}X_{ik}X_{\text{is}}X_{it}\right)}_{c}\\ +2\left(\frac{1}{T}\sum_{i=1}^{T}X_{ij}X_{ik}\right)\left(\frac{1}{T}\sum_{i=1}^{T}X_{\text{is}}X_{it}\right)\cdot \frac{1}{T-1}\sum_{i=1}^{T}{\left(X_{ij}X_{ik}\right)}_{c}{\left(X_{\text{is}}X_{it}\right)}_{c}. \end{array}$$

Let ℛ_*αl*_ be the rejection region for the *l*th hypothesis with certain level *α*, then the corresponding *p* value can be determined as

$$p_{l}=\underset{\alpha }{\inf}\;P_{0l}\left(\gamma_{jk,st}\in {ℛ}_{\alpha l}\right).$$

Recall that we have established the asymptotic normal distribution for the sample moment estimator ${\hat{\Theta }}_{jk,st}$ in Equation 8. Thus, the value *p*_*l*_ can be specifically computed based on the normal distribution with a certain level *α*.

Under the multiple testing setting, we now aim to control the FDR (Benjamini & Hochberg, 1995), defined as

$$\text{FDR}=E\left[\frac{\#\;\text{false}\;\text{positives}}{\#\;\text{rejections}}\right],$$

under some predetermined level *α*. Given that the *p* values *p*_*l*_s for different edge-pair connections can be dependent, we adopt the Benjamini–Yekutieli procedure (Benjamini & Yekutieli, 2001) as summarized in Algorithm 1, which controls FDR among dependent hypotheses with a cutoff value for ordered *p* values.


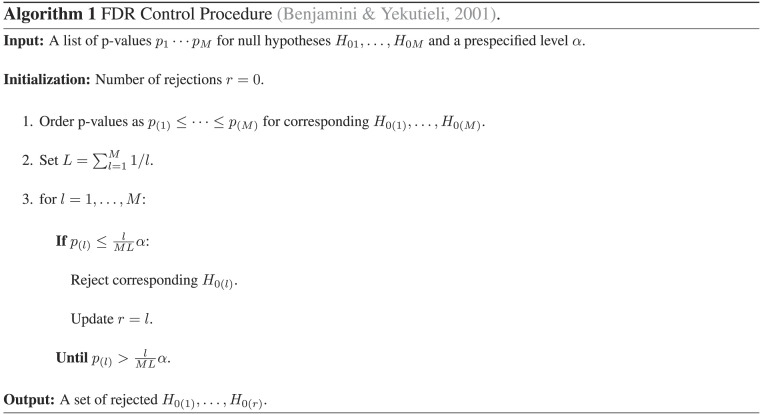


### Thresholded Estimator of ceFC

In the Results section for the sample moment estimator, we see that its estimation error fails to converge to zero when the number of brain parcels *N* is greater than the sample size *T*.

To address this issue, we propose a thresholded estimator by truncating entries to zero if their absolute values are below a specified threshold for a class of sparse ceFC matrices, thus providing a consistent estimation even when the number of parcels is greater than the number of time frames. This thresholding procedure is a necessary statistical step to mitigate the risk of overfitting and to ensure that subsequent analyses are based on reliable signals rather than spurious correlations.

We first introduce the following notations. For an *N* × *N* matrix **A** = [*a*_*ij*_] ∈ ℝ^*N*×*N*^, we define the thresholding operator **T**_*τ*_ as:

$$T_{\tau }\left(A\right)=\left[a_{ij}1\left(\left|a_{ij}\right|\geq \tau \right)\right].$$

With this operator **T***_τ_*, we truncate the entries *a*_*ij*_ in a matrix **A** to zero if their absolute values are less than a certain cutoff value *τ*.

We further consider the class of sparse ceFC matrices that

$$U_{\tau }\left(m\right)=\left\{\Theta :\sum_{\left\{j,k,s,t\right\}}1\left(\Theta_{jk,st}\neq 0\right)\leq m\right\}.$$

That is, the number of nonzero entries in such sparse ceFC matrices would be no more than certain values *m*. For such a sparse ceFC matrix **Θ**, we propose to estimate each entry Θ_*jk*,*st*_ by its thresholded estimator

$$T_{\tau }\left({\hat{\Theta }}_{jk,st}\right).$$ (11)

### Comparison of Sample Moment Estimation With Thresholded Estimation

In the previous discussion, we showed the statistical rate of the sample moment estimator and the thresholded estimator under the Frobenius norm. We can see that in situations where the true ceFC is sparse under the class $U_{\tau }\left(m\right)$, the thresholded estimator (Equation 11) approaches the true centered eFC matrix with an upper bound delineated by

$$\sqrt{\frac{m\log^{4}\left({TN}^{4}\right)}{T}},$$

which converges to zero even for *N* > *T*. On the other hand, the sample moment estimator (Equation 6) converges to the true centered eFC matrix under the bound as the function

$$\sqrt{\frac{N^{4}\log^{4}\left({TN}^{4}\right)}{T}},$$

failing to converge to zero when *N* > *T*. Accordingly, the proposed thresholded estimator provides a consistent estimate for centered eFC in the high-dimensional scenario where *N* > *T*. Moreover, as shown in the Results section, even under the classical setting with a sufficient sample size *T*, the thresholded estimator approaches the true centered eFC matrix under a substantially smaller bound with an appropriate thresholding value *τ*, in comparison with the sample moment estimator.

### Simulation Study

#### Simulation settings for estimation evaluation.

In the simulation study for estimation evaluation, we generate *T* independent and identically distributed *N*-dimensional vectors from a multivariate normal distribution, that is, ***X****_i_* ∼ *N*(**0**, **Σ**), where **Σ** is some *N* × *N* covariance matrix generated from the MSC dataset, which will be specified later in this section. Under the Gaussian assumption, the true ceFC entry is evaluated as $\Theta_{jk,st}*=\sigma_{js}\sigma_{kt}+\sigma_{jt}\sigma_{ks}$ for {*jk*, *st*} ∈ {1, …, *N*(*N* −1)/2} by Isserlis’s theorem (Isserlis, 1918), where *σ_jk_* denotes the covariance between node *j* and node *k*. We consider both nonsparse and sparse covariance matrices **Σ**. The resulting matrix ***X*** ∈ ℝ^*T*×*N*^ can be viewed as the synthetic BOLD signals, where the number of rows *T* represents the number of time frames, and the number of variables *N* denotes the number of parcels.

We start with the nonsparse scenario for covariance **Σ**. Specifically, we calculate the sample covariance matrix for each scan/subject of the MSC resting-state fMRI data (Gordon et al., 2017) and set **Σ** to be the average of the computed sample covariance matrices. This yields a **Σ** ∈ ℝ^200×200^ matrix with 200 brain parcels using Schaefer200 parcellation (Schaefer et al., 2018), which can be further partitioned into 16 brain systems by Yeo et al. (2011). We also consider a subset of the brain with *N* = 50, where we randomly select 25% of the brain regions in each system. Under the Gaussian distribution, the true ceFC entry between edge *jk* and edge *st* (Equation 5) is evaluated as $\Theta_{jk,st}*=\sigma_{js}\sigma_{kt}+\sigma_{jt}\sigma_{ks}$ for {*jk*, *st*} ∈ {1, …, *N*(*N* − 1)/2} by Isserlis’s theorem (Isserlis, 1918).

For the sparse scenario, we truncate the absolute values of the above matrix **Σ** below 0.3 to 0, resulting in 97% sparsity for the 50 × 50 covariance matrix and 92% sparsity for the 200 × 200 covariance matrix. Finally, to ensure that the sparse covariance matrix **Σ** is positive definite, we set Σ*_jj_* = |Λ_min_(**Σ**)| + 0.01 for *j* = 1, …, *N*, where Λ_min_(**Σ**) is the minimum eigenvalue of **Σ** (Tan, Lu, Zhang, & Liu, 2021). We then rescale **Σ** to obtain a correlation matrix such that all variables are on the same scale with variance one.

#### Simulation settings for split-half reliability.

To formally assess the empirical robustness of eFC and ceFC, we implement a split-half reliability analysis across 100 simulation trials. The primary goal of this analysis was to quantify how reliably each method could estimate higher-order connectivity. Based on a similar setting mentioned above, we split the time series into first and second halves, with *T* = 500 for each half. The eFC and ceFC matrices are computed separately for each half of the data. To determine the reliability for each edge pair, we aggregate the results across 100 trials to form two vectors, where the first vector contains the estimated eFC or ceFC values from the first half, and the second vector contains the corresponding values from the second half. The reliability score for that specific edge pair is then defined as the correlation between these two vectors. This process yields a full distribution of reliability scores for all edge pairs, allowing for a direct comparison of estimation robustness between eFC and ceFC.

#### Estimation evaluation.

To compare the performance across the two methods, we first compute the estimation error between the proposed estimator $\hat{\Theta }=\left[{\hat{\Theta }}_{jk,st}\right]$ and the true ceFC matrix $\Theta *=\left[\Theta_{jk,st}\right]$ under the Frobenius norm ${\left\|\hat{\Theta }-\Theta *\right\|}_{F}=\sqrt{\sum_{jk,st}{\left({\hat{\Theta }}_{jk,st}-\Theta_{jk,st}*\right)}^{2}}$ for all {*jk*, *st*} ∈ {1, …, *N*(*N* −1)/2}. Then, we assess the estimation accuracy of each estimator using the relative Frobenius norm error. This metric quantifies the estimation error relative to the magnitude of the true underlying signal, providing a standardized measure of performance that is comparable across different simulation parameters. It is defined as the Frobenius norm of the difference between the estimated matrix ($\hat{\Theta }$) and the true matrix (Θ*), normalized by the Frobenius norm of the true matrix:

$$\frac{{\left\|\hat{\Theta }-\Theta *\right\|}_{F}}{{\left\|\Theta ,*\right\|}_{F}}$$

Moreover, we define the TPR and FPR as the proportion of correctly estimated nonzero entries and incorrectly estimated nonzero entries, respectively:

$$\text{TPR}\left(\hat{\Theta },\Theta *\right)=\frac{\#\left\{\left(jk,st\right):{\hat{\Theta }}_{jk,st}\neq 0\;\text{and}\;\Theta_{jk,st}*\neq 0\right\}}{\#\left\{\left(jk,st\right):\Theta_{jk,st}*\neq 0\right\}};$$ (12)

$$\text{FPR}\left(\hat{\Theta },\Theta *\right)=\frac{\#\left\{\left(jk,st\right):{\hat{\Theta }}_{jk,st}\neq 0\;\text{and}\;\Theta_{jk,st}*=0\right\}}{\#\left\{\left(jk,st\right):\Theta_{jk,st}*=0\right\}}.$$ (13)

#### Simulation settings for inference framework.

The simulation setting for the inference framework is similar to the process in the Simulation Settings for Estimation Evaluation section, except that we generate a sparse ceFC matrix with moderate signals. To achieve this, we adopt the approach in Tan et al. (2021) by generating a sparse covariance matrix with some nonzero values. Specifically, we randomly assign 0.9 to 5% and 2% of off-diagonal entries in an identity matrix for the *N* = 50 and *N* = 200 cases, respectively. We then employ the same procedure as mentioned earlier to ensure that the resulting sparse covariance matrix remains positive definite.

### Clustering Procedure With Overlapping Metrics

Motivated by Faskowitz et al. (2020), we perform spectral clustering on each of the aforementioned three ceFC estimators in the Identifying Overlapping Communities section to identify overlapping communities of the brain regions. That is, we conduct eigen decomposition on each of the ceFC estimators, extract 50 eigenvectors with the highest eigenvalues, and subsequently perform *k-*means clustering with *k* = 5 on the resulting eigenvectors with 5 communities for visualization purposes.

To obtain overlapping communities for the brain regions, we project the edge communities back to the brain region level following the procedure in Faskowitz et al. (2020). Specifically, for each brain region, we calculate the proportion of edges in each of the estimated five communities, allowing each brain region to belong to multiple communities with different weights. We note that cluster labels from the three different estimators can be different. To address this issue, we relabel the cluster labels by maximizing the pairwise Matthews correlation coefficients between a pair of estimators (Matthews, 1975). That is, we relabel the community labels for each estimator using the above method by matching them to the average thresholded estimator.

### Sensitivity Analysis

In this section, we implement a sensitivity analysis to compare the stability of the three types of estimators regarding the hypothesis testing (Equation 4) on the community proportions obtained from each type of estimator. We consider the test results from each average estimator, denoted as *μ*^avg^, as ground truth. Across the subject-specific test outcome, denoted as *μ*^sub^, we define the FPR and the FNR as the proportion of communities *c* or systems *s* incorrectly identified as significant and the proportion of communities *c* or systems *s* incorrectly identified as insignificant across all communities, respectively:

$$\begin{array}{ll} \text{FPR}\left(\mu^{\text{sub}},\mu^{\text{avg}}\right) & =\frac{\#\left\{\left(c,s\right):\mu_{cs}^{\text{sub}}>0\;\text{and}\;\mu_{cs}^{\text{avg}}=0\right\}}{\#\left\{\left(c,s\right):\mu_{cs}^{\text{avg}}=0\right\}};\\ \text{FNR}\left(\mu^{\text{sub}},\mu^{\text{avg}}\right) & =\frac{\#\left\{\left(c,s\right):\mu_{cs}^{\text{sub}}=0\;\text{and}\;\mu_{cs}^{\text{avg}}>0\right\}}{\#\left\{\left(c,s\right):\mu_{cs}^{\text{avg}}>0\right\}}. \end{array}$$ (14)

## ACKNOWLEDGMENTS

R.F.B. acknowledges support from the National Science Foundation (NSF; NCS-FO Award 2023985). K.M.T. acknowledges support from the NSF (CAREER DMS-2238428).

## SUPPORTING INFORMATION

Supporting information for this article is available at https://doi.org/10.1162/NETN.a.570.

## AUTHOR CONTRIBUTIONS

Junting Wang: Conceptualization; Data curation; Formal analysis; Investigation; Methodology; Software; Validation; Visualization; Writing – original draft; Writing – review & editing. Youngheun Jo: Conceptualization; Investigation; Methodology; Supervision; Validation; Visualization; Writing – review & editing. Junwei Lu: Conceptualization; Methodology; Supervision; Writing – review & editing. Richard Betzel: Conceptualization; Methodology; Supervision; Writing – review & editing. Ji Zhu: Methodology; Supervision; Writing – review & editing. Kean Ming Tan: Conceptualization; Investigation; Methodology; Project administration; Supervision; Validation; Visualization; Writing – review & editing.

## DATA AVAILABILITY

MSC data are available on OpenNeuro at https://openneuro.org/datasets/ds000224/versions/1.0.1.

## CODE AVAILABILITY

Code for ceFC and its related computation is available at https://github.com/Junting-Wang/centered-eFC.git.

## Supplementary Material



## TECHNICAL TERMS

Functional connectivity: The pairwise interaction between brain regions, often computed as the correlation between fMRI BOLD activities of a pair of brain regions. Denoted as the node functional connectivity.
Edge-centric functional connectivity: The scaled cofluctuation of two edge time series.
Edge time series: The cofluctuations of fMRI BOLD activities of a pair of brain regions over time, which can be obtained by their element-wise product.
False discovery rate: The expected ratio of the number of false positives to the total number of rejections.
Thresholding: A statistical technique truncates all elements to zero if their absolute values fall below a specified level, while leaving all other elements unchanged.
Centered edge-centric functional connectivity: The unbiased covariation of two edge time series after centering.
Type I error: Incorrect rejection of a true null hypothesis, also known as a false positive.
Power: The probability that a test correctly rejects the null hypothesis when the alternative hypothesis is true.
Spectral clustering: A clustering method that uses eigenvalues and eigenvectors of the similarity matrix of the data to project into lower dimensions space and then perform clustering.
