# Supplementary material for: Higher-order statistics for constructing centered edge functional connectivity
Source: Netw Neurosci. 2026 Jul 27;10(3):706–37. doi: 10.1162/NETN.a.570 (PMC13418521; doi:10.1162/NETN.a.570)
Supplement: Supplementary file 1 [file netn-10-3-706-s001.pdf]

Journal: **NETWORK NEUROSCIENCE**

SUPPORTING INFORMATION

Supporting Information for Higher-Order Statistics for Constructing Centered Edge Functional Connectivity

Junting Wang, Youngheun Jo, Junwei Lu, Richard Betzel, Ji Zhu, Kean Ming Tan

Supplementary Results

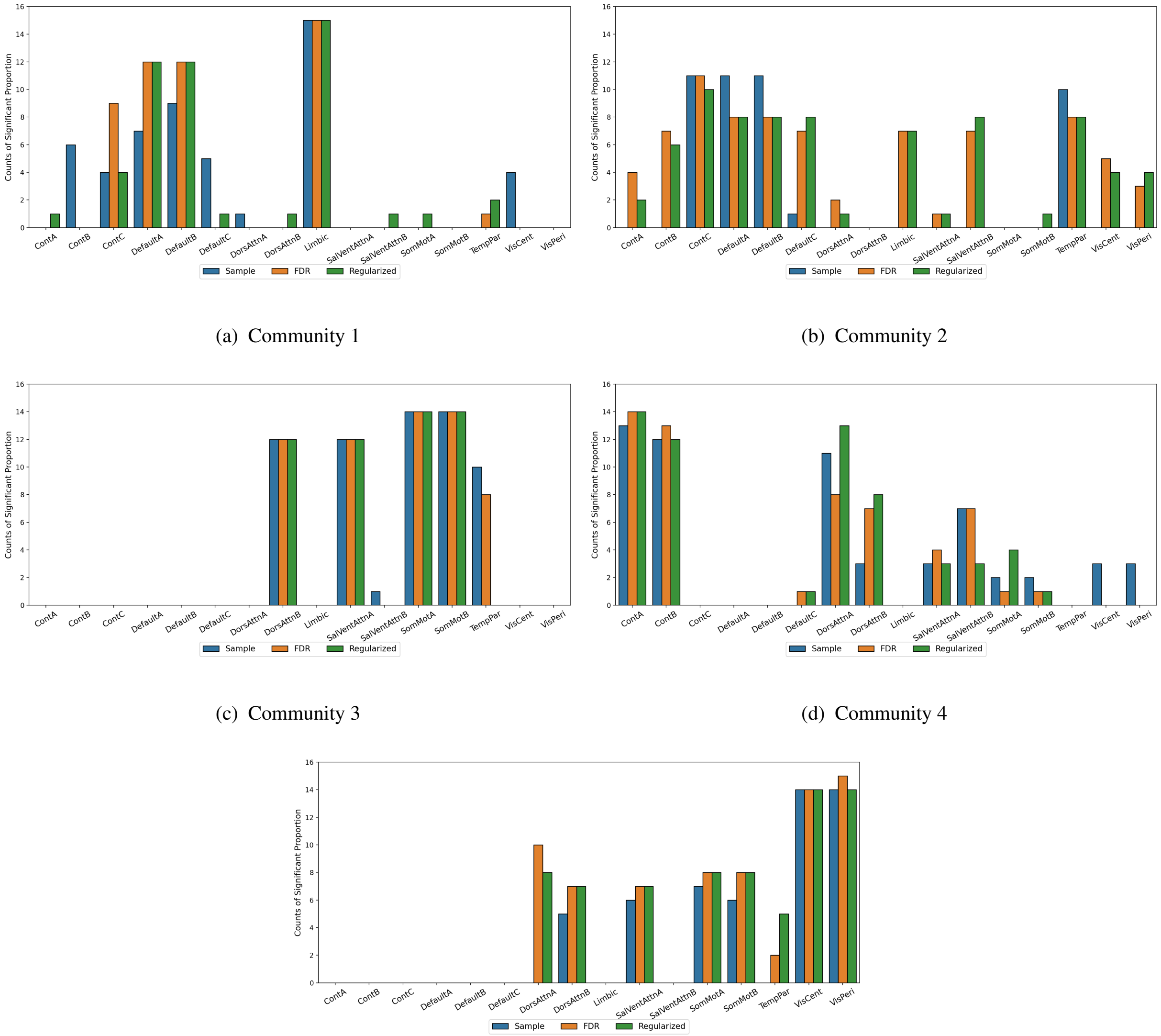

(a) Community 1

(b) Community 2

(c) Community 3

(d) Community 4

(e) Community 5

Figure S1. Counts of significant outcomes from pairwise comparisons of edge proportions among brain systems. The top three brain systems, identified by their top three highest counts within each method, are highlighted in Figure 4.

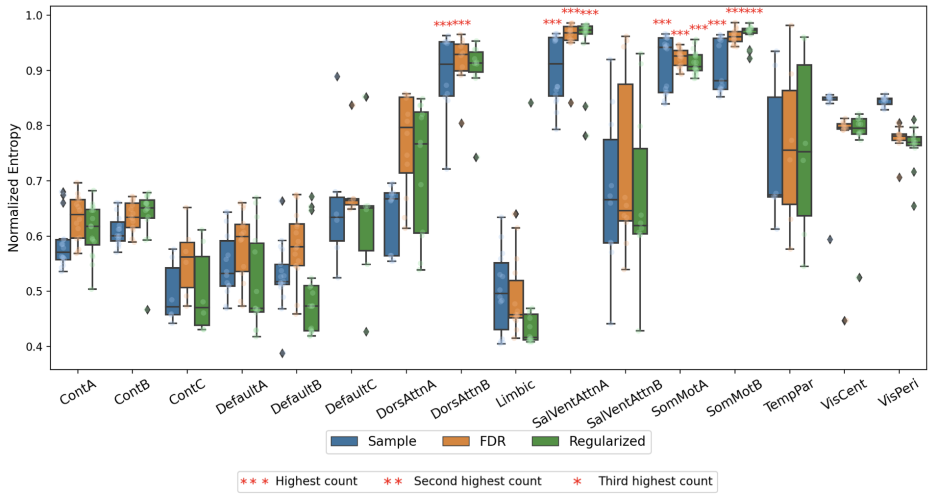

Figure S2. Normalized entropy of edge proportions in Figure 3 over all communities. The plot highlights the top three brain systems that demonstrate a significantly greater normalized entropy of edges in pairwise comparisons with other systems. $***$ denotes the system with the highest count of significant outcomes, $**$ represents the second highest, and $*$ signifies the third highest.

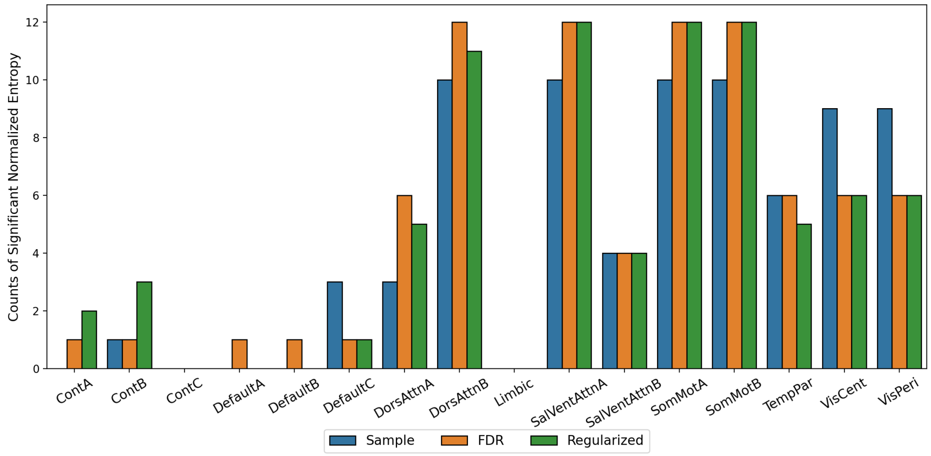

Figure S3. Counts of significant outcomes from pairwise comparisons of normalized entropy among brain systems. The top three brain systems, identified by their top three highest counts within each method, are highlighted in Supplementary Figure **??**.

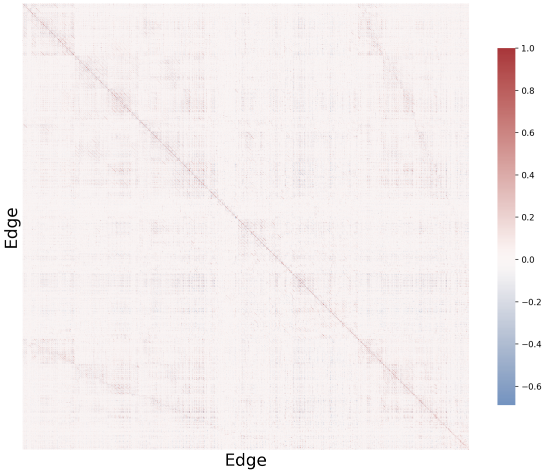

(a) eFC

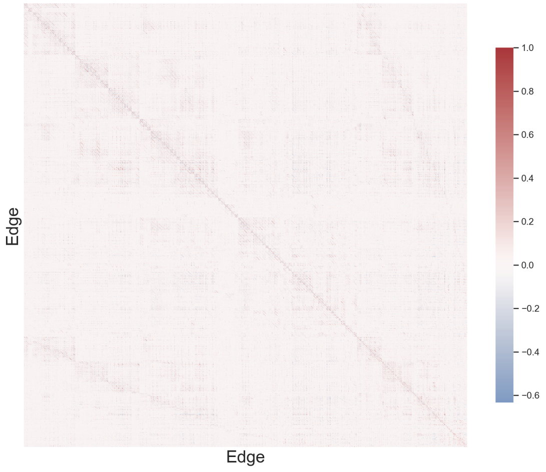

(b) ceFC

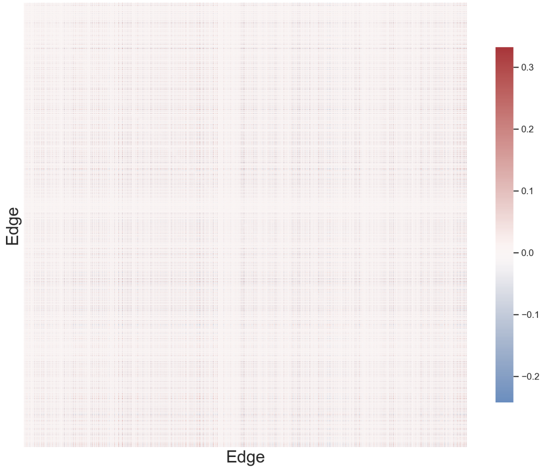

(c) eFC - ceFC

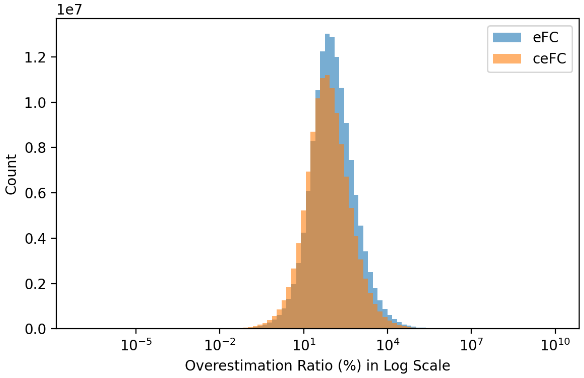

(d) Overestimation Ratio

Figure S4. Sample moment estimation of eFC, ceFC, and eFC − ceFC in (a), (b), and (c), respectively. Plot (d) shows the distribution of the overestimation ratio (%) on a log scale.

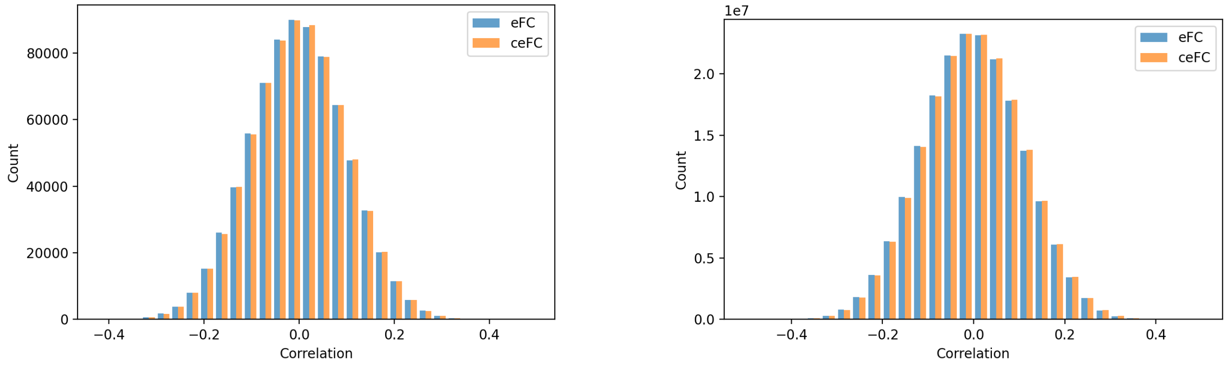

(a) $N = 50$ (b) $N = 200$

Figure S5. Correlation of each method from two halves of the simulation as split-half reliability. Plots (a) and (b) show the count of edge pairs for corresponding correlation values with $N = 50$ and $N = 200$ parcels, respectively.

Technical Lemmas with proofs for the consistency results for sample moment estimator

Under the sub-Gaussian Condition, we study the statistical rate of the sample moment estimator under the Frobenius norm. To see this, we start with the upper bound of the max norm

$$\max_{\{j,k,s,t\}\in\{1,\dots,N\}^4} \left|\widehat{\Theta}_{jk,st} - \Theta_{jk,st}\right|,$$

which consists of the second-order and fourth-order moments in the Lemma **??** and Lemma **??**, respectively.

Lemma 1. Under the sub-Gaussian condition as discussed in Null Hypothesis section, we have

$$\max_{\{j,k,s,t\}\in\{1,\dots,N\}^4} \left|\frac{1}{T}\sum_{i=1}^{T} X_{ij}X_{ik} \cdot \frac{1}{T}\sum_{i=1}^{T} X_{is}X_{it} - \sigma_{jk}\sigma_{st}\right| \le C_1\sqrt{\frac{\log(TN)}{T}} + C_2\frac{\log(TN)}{T},$$

for some constants C_1 and C_2, with probability at least $1 - T^{-1}$.

Proof. We start the proof with decomposition of the quantity $\left|T^{-1}\sum_{i=1}^{T} X_{ij}X_{ik} \cdot T^{-1}\sum_{i=1}^{T} X_{is}X_{it} - \sigma_{jk}\sigma_{st}\right|$ by adding and subtracting terms:

$$\begin{aligned}
&\left|\frac{1}{T}\sum_{i=1}^{T} X_{ij}X_{ik} \cdot \frac{1}{T}\sum_{i=1}^{T} X_{is}X_{it} - \sigma_{jk}\sigma_{st}\right| \\
&= \left|\left(\frac{1}{T}\sum_{i=1}^{T} X_{ij}X_{ik} - \sigma_{jk}\right)\left(\frac{1}{T}\sum_{i=1}^{T} X_{is}X_{it}\right)\right. \\
&\quad \left. +\sigma_{jk}\left(\frac{1}{T}\sum_{i=1}^{T} X_{is}X_{it} - \sigma_{st}\right)\right| \\
&\le \underbrace{\left|\left(\frac{1}{T}\sum_{i=1}^{T} X_{ij}X_{ik} - \sigma_{jk}\right)\left(\frac{1}{T}\sum_{i=1}^{T} X_{is}X_{it} - \sigma_{st}\right)\right|}_{I} \\
&\quad + \underbrace{\left|\left(\frac{1}{T}\sum_{i=1}^{T} X_{ij}X_{ik} - \sigma_{jk}\right)\sigma_{st}\right|}_{II} \\
&\quad + \underbrace{\left|\sigma_{jk}\left(\frac{1}{T}\sum_{i=1}^{T} X_{is}X_{it} - \sigma_{st}\right)\right|}_{III}.
\end{aligned}$$

It suffices to show that I, II, and III are upper bounded by $C_1\sqrt{T^{-1}\log(TN)} + C_2T^{-1}\log(TN)$ for some constants C_1, C_2.

By an application of Lemma 1 from **?**, for some constant C, we have

$$II \leq C\sqrt{\frac{\log(T)}{T}},$$

with probability at least $1 - T^{-1}$. By union bound, we further obtain

$$\max_{\{j,k,s,t\}\in\{1,\ldots,N\}^4} II \leq C_1\sqrt{\frac{\log(TN)}{T}},$$

with probability at least $1 - T^{-1}$ for some constant C_1. We obtain the same result for III.

For I, note that

$$\max_{\{j,k,s,t\}\in\{1,\ldots,N\}^4} I \leq \max_{\{j,k,s,t\}\in\{1,\ldots,N\}^4}\left(\frac{II}{|\sigma_{st}|}\frac{III}{|\sigma_{jk}|}\right).$$

By union bound, for some constant C', we have

$$\begin{aligned}
&\mathbb{P}\left(\max_{\{j,k,s,t\}\in\{1,\ldots,N\}^4}\left(\frac{II}{|\sigma_{st}|}\frac{III}{|\sigma_{jk}|}\right) \geq C'^2\frac{\log(TN)}{T}\right)\\
&\leq \mathbb{P}\left(\left\{\max_{\{j,k,s,t\}\in\{1,\ldots,N\}^4}\frac{II}{|\sigma_{st}|} \geq C'\sqrt{\frac{\log(TN)}{T}}\right\}\right.\\
&\qquad\left.\bigcup\left\{\max_{\{j,k,s,t\}\in\{1,\ldots,N\}^4}\frac{III}{|\sigma_{jk}|} \geq C'\sqrt{\frac{\log(TN)}{T}}\right\}\right)\\
&\leq \mathbb{P}\left(\max_{\{j,k,s,t\}\in\{1,\ldots,N\}^4}\frac{II}{|\sigma_{st}|} \geq C'\sqrt{\frac{\log(TN)}{T}}\right)\\
&\qquad+\mathbb{P}\left(\max_{\{j,k,s,t\}\in\{1,\ldots,N\}^4}\frac{III}{|\sigma_{jk}|} \geq C'\sqrt{\frac{\log(TN)}{T}}\right)\\
&\leq \frac{2}{T}.
\end{aligned}$$

Therefore, for some constant C_2,

$$\max_{\{j,k,s,t\}\in\{1,\ldots,N\}^4} I \leq C_2\frac{\log(TN)}{T}$$

with probability at least $1 - 2T^{-1}$.

Combining the aforementioned upper bounds on I, II, and III, we obtain the desired results. □

It remains to find the concentration inequality for the fourth-order moment part, which is summarized in the following lemma.

Lemma 2. Under the sub-Gaussian condition as discussed in Null Hypothesis section, we have

$$\max_{\{j,k,s,t\}\in\{1,\cdots,N\}^4}\left|\frac{1}{T}(\sum_{i=1}^{T} X_{ij}X_{ik}X_{is}X_{it} - \mathbb{E}[X_{ij}X_{ik}X_{is}X_{it}])\right| \leq C_3\sqrt{\frac{\log^4(TN^4)}{T}},$$

for some constant C_3 with probability at least $1 - T^{-1}$.

Proof. We aim to find the bound u, so that

$$\mathbb{P}\left(\max_{\substack{\{j,k,s,t\}\\ \in\{1,\dots,N\}^4}}\left|\frac{1}{T}(\sum_{i=1}^{T} X_{ij}X_{ik}X_{is}X_{it} - \mathbb{E}[X_{ij}X_{ik}X_{is}X_{it}])\right| > u\right) < \frac{1}{T}.$$

We start with union bound,

$$\begin{aligned}
&\mathbb{P}\left(\max_{\substack{\{j,k,s,t\}\\ \in\{1,\dots,N\}^4}}\left|\frac{1}{T}\sum_{i=1}^{T} X_{ij}X_{ik}X_{is}X_{it} - \mathbb{E}[X_{ij}X_{ik}X_{is}X_{it}]\right| > u\right)\\
&= \mathbb{P}\left(\bigcup_{\substack{\{j,k,s,t\}\\ \in\{1,\dots,N\}^4}}\left|\frac{1}{T}\sum_{i=1}^{T} X_{ij}X_{ik}X_{is}X_{it} - \mathbb{E}[X_{ij}X_{ik}X_{is}X_{it}]\right| > u\right)\\
&\leq \sum_{\substack{\{j,k,s,t\}\\ \in\{1,\dots,N\}^4}} \mathbb{P}\left(\left|\sum_{i=1}^{T} X_{ij}X_{ik}X_{is}X_{it} - \mathbb{E}[X_{ij}X_{ik}X_{is}X_{it}]\right| > Tu\right)\\
&\leq \sum_{\substack{\{j,k,s,t\}\\ \in\{1,\dots,N\}^4}} \frac{\mathbb{E}\left[\left(\sum_{i=1}^{T} X_{ij}X_{ik}X_{is}X_{it} - \mathbb{E}[X_{ij}X_{ik}X_{is}X_{it}]\right)^v\right]}{(Tu)^v}
\end{aligned}$$

where the last inequality follows from an application of Markov inequality.

It suffices to obtain the upper bound of $\mathbb{E}[(\sum_{i=1}^{T} X_{ij}X_{ik}X_{is}X_{it} - \mathbb{E}[X_{ij}X_{ik}X_{is}X_{it}])^v]$. Note that by the product of sum, we can rewrite the expectation as

$$\begin{aligned}
&\mathbb{E}\left[\left(\sum_{i=1}^{T} X_{ij}X_{ik}X_{is}X_{it} - \mathbb{E}[X_{ij}X_{ik}X_{is}X_{it}]\right)^v\right] \\
&= \sum_{i_1,\ldots,i_T\in[T]} \mathbb{E}\left[\prod_{q=1}^{v}\left(X_{ij_q}X_{ik_q}X_{is_q}X_{it_q}\right.\right. \\
&\qquad \left.\left. - \mathbb{E}[X_{ij_q}X_{ik_q}X_{is_q}X_{it_q}]\right)\right] \\
&= \sum_{v_1+\ldots+v_T=v/2} \prod_{i=1}^{T} \mathbb{E}\left(X_{ij_q}X_{ik_q}X_{is_q}X_{it_q}\right. \\
&\qquad \left. - \mathbb{E}[X_{ij_q}X_{ik_q}X_{is_q}X_{it_q}]\right)^{2v_i} \\
&\leq \sum_{v_1+\ldots+v_T=v/2} \prod_{i=1}^{T} \mathbb{E}\left(|X_{ij_q}X_{ik_q}X_{is_q}X_{it_q}|\right. \\
&\qquad \left. + |\mathbb{E}[X_{ij_q}X_{ik_q}X_{is_q}X_{it_q}]|\right)^{2v_i} \\
&= \sum_{v_1+\ldots+v_T=v/2} \prod_{i=1}^{T} \mathbb{E}\left(\sum_{m=0}^{2v_i} \binom{2v_i}{m} |X_{ij_q}X_{ik_q}X_{is_q}X_{it_q}|^{2v_i-m}\right. \\
&\qquad \left. \cdot |\mathbb{E}[X_{ij_q}X_{ik_q}X_{is_q}X_{it_q}]|^m\right),
\end{aligned}$$

where the inequality is obtained by the triangle inequality and the last equality is obtained by rewriting the product of sum.

By Cauchy-Schwarz inequality, we have

$$\begin{aligned}
&|\mathbb{E}[X_{ij_q}X_{ik_q}X_{is_q}X_{it_q}]|^m \\
&\leq (\mathbb{E}[X_{ij_q}^2 X_{ik_q}^2])^{m/2}(\mathbb{E}[X_{is_q}^2 X_{it_q}^2])^{m/2} \\
&\leq (\mathbb{E}[X_{ij_q}^4]\mathbb{E}[X_{ik_q}^4]\mathbb{E}[X_{is_q}^4]\mathbb{E}[X_{it_q}^4])^{m/4}(\mathbb{E}[X_{is_q}^2 X_{it_q}^2])^{m/2} \\
&\leq K^{4m}4^{2m},
\end{aligned} \tag{S.1}$$

for some constant K, where the last inequality is obtained by the sub-Gaussian condition.

Applying the same process for $\mathbb{E}(|X_{ij_q}X_{ik_q}X_{is_q}X_{it_q}|^{2p_i-m})$, we have

$$
\begin{aligned}
&\sum_{v_1+\ldots+v_T=v/2}\prod_{i=1}^{T}\mathbb{E}\Bigg(\sum_{m=0}^{2v_i}\binom{2v_i}{m}\Big|X_{ij_q}X_{ik_q}X_{is_q}X_{it_q}\Big|^{2v_i-m} \\
&\quad\cdot\Big|\mathbb{E}[X_{ij_q}X_{ik_q}X_{is_q}X_{it_q}]\Big|^{m}\Bigg) \\
&\le \sum_{v_1+\ldots+v_T=v/2}\prod_{i=1}^{T}\sum_{m=0}^{2v_i}\binom{2v_i}{m}\Big(\mathbb{E}[X_{ij_q}^{4v_i-2m}X_{ik_q}^{4v_i-2m}]\Big)^{1/2} \\
&\quad\cdot\Big(\mathbb{E}[X_{is_q}^{4v_i-2m}X_{it_q}^{4v_i-2m}]\Big)^{1/2}\cdot K^{4m}4^{2m} \\
&\le \sum_{v_1+\ldots+v_T=v/2}\prod_{i=1}^{T}\sum_{m=0}^{2v_i}\binom{2v_i}{m}\Big(\mathbb{E}[X_{ij_q}^{8v_i-4m}]\mathbb{E}[X_{ik_q}^{8v_i-4m}] \\
&\quad\cdot\mathbb{E}[X_{is_q}^{8v_i-4m}]\mathbb{E}[X_{it_q}^{8v_i-4m}]\Big)^{1/4}\cdot K^{4m}4^{2m} \\
&\le \sum_{v_1+\ldots+v_T=v/2}\prod_{i=1}^{T}\sum_{m=0}^{2v_i}\binom{2v_i}{m}\Big[K^{(8v_i-4m)4}(8v_i-4m)^{(4v_i-2m)4}\Big]^{1/4} \\
&\quad\cdot K^{4m}4^{2m} \\
&= \sum_{v_1+\ldots+v_T=v/2}\prod_{i=1}^{T}\sum_{m=0}^{2v_i}\binom{2v_i}{m}K^{8v_i}4^{4v_i}(2v_i-m)^{4v_i-2m} \\
&\le (Cv^4T)^{v/2},
\end{aligned}
$$

for some constant C, where the last inequality holds by an application of Lemma **??**.

Choose $v=\log(TN^4)$, $u=C_3\sqrt{\log^4(TN^4)/T}$ and we obtain the desired results. □

Combining the results for the second- and fourth-order moments, we have the following result for the the sample moment estimator of ceFC.

Lemma 3. Under the sub-Gaussian condition as discussed in Null Hypothesis section, we have

$$
\begin{aligned}
\max_{\{j,k,s,t\}\in\{1,\ldots,N\}^4}\Big|\widehat{\Theta}_{jk,st}-\Theta_{jk,st}\Big| &\le C_1\sqrt{\frac{\log(TN)}{T}}+C_2\frac{\log(TN)}{T} \\
&\quad + C_3\sqrt{\frac{\log^4(TN^4)}{T}},
\end{aligned}
$$

for some constants C_1, C_2, C_3, with probability at least $1-T^{-1}$.

Proof. By triangle inequality, we decompose the quantity:

$$\left|\widehat{\Theta}_{jk,st} - \Theta_{jk,st}\right| \leq \left|\frac{1}{T}\sum_{i=1}^{T} X_{ij}X_{ik}X_{is}X_{it} - \mathbb{E}[X_jX_kX_sX_t]\right| + \left|\frac{1}{T}\sum_{i=1}^{T} X_{ij}X_{ik} \cdot \frac{1}{T}\sum_{i=1}^{T} X_{is}X_{it} - \sigma_{jk}\sigma_{st}\right|.$$

38 Applying Lemma **??** and **??**, we get the desired result. □

39 ***Proof of the consistency results for sample moment estimator***

Proof. By Lemma **??**, we have

$$|\widehat{\Theta}_{jk,st} - \Theta^{\star}_{jk,st}| \leq \max_{\{j,k,s,t\}} |\widehat{\Theta}_{jk,st} - \Theta^{\star}_{jk,st}| \leq C'\sqrt{\frac{\log^4(TN^4)}{T}},$$

40 for some constant C' with probability at least $1 - T^{-1}$.

Then we can write

$$\begin{aligned} &\|\widehat{\Theta}_{jk,st} - \Theta_{jk,st}\|_F^2 \\ =& \sum_{\{j,k,s,t\}} (\widehat{\Theta}_{jk,st} - \Theta_{jk,st})^2 \\ \leq& \sum_{\{j,k,s,t\}} \max_{\{j,k,s,t\}} (\widehat{\Theta}_{jk,st} - \Theta_{jk,st})^2 \\ \leq& CN^4\frac{\log^4(TN^4)}{T}, \end{aligned}$$

41 for some constant C with probability at least $1 - T^{-1}$. □

42 ***Proof of FDR Control Procedure***

43 *Proof.* By law of large number and Slutsky's theorem, the sample estimate $\widehat{\Lambda}_{jk,st} \xrightarrow{p} \Lambda_{jk,st}$. Based on
44 the asymptotic normality of $\widehat{\Theta}_{jk,st}$, we further obtain $\gamma_{jk,st} \xrightarrow{d} \mathcal{N}(0,1)$ as $n \to \infty$.

For any $\alpha \in (0,1)$, the event $\{p_{(l)} \leq \alpha\}$ is equivalent to the event $\{|\gamma_{jk,st}| \geq z_{\alpha/2}\}$. As $\gamma_{jk,st} \xrightarrow{d} \mathcal{N}(0,1)$, $\mathbb{P}(|\gamma_{jk,st}| \geq z_{\alpha/2}) \longrightarrow \alpha$. We obtain

$$\mathbb{P}(p_{(l)} \leq \alpha) = \mathbb{P}(|\gamma_{jk,st}| \geq z_{\alpha/2}) = \alpha + o(1). \tag{S.2}$$

For a set of M hypotheses, denote $\mathcal{H}_0$ as the set of true null hypotheses and R as the number of rejections. By the procedure of FDR control in **?**, we have

$$R = \sum_{l=1}^{M} \mathbb{1}\left(p_l \le \frac{l}{ML}\alpha\right),$$

where $L = \sum_{l=1}^{M} 1/l$. Following the proof of Theorem 4.2 in **?**, we have

$$\frac{1}{R} = \sum_{r=R}^{\infty} \frac{1}{r(r+1)} = \sum_{r=1}^{\infty} \frac{\mathbb{1}(r \ge R)}{r(r+1)},$$

and

$$\begin{aligned}
\text{FDR} &= \sum_{l\in\mathcal{H}_0} \mathbb{E}\left[\frac{\mathbb{1}\left(p_l \le \frac{R}{ML}\alpha\right)\mathbb{1}(R>0)}{R}\right] \\
&= \sum_{r=1}^{\infty} \frac{\mathbb{1}(r \ge R)}{r(r+1)} \sum_{l\in\mathcal{H}_0} \mathbb{E}\left[\mathbb{1}\left(p_l \le \frac{R}{ML}\alpha\right)\mathbb{1}(R>0)\right] \\
&\le \sum_{r=1}^{\infty} \frac{1}{r(r+1)} \sum_{l\in\mathcal{H}_0} \mathbb{P}\left(p_l \le \frac{\min(r,M)}{ML}\alpha\right).
\end{aligned}$$

By (**??**), we further obtain

$$\begin{aligned}
\text{FDR} &\le \sum_{r=1}^{\infty} \frac{1}{r(r+1)} \sum_{l\in\mathcal{H}_0} \left(\frac{\min(r,M)}{ML}\alpha + o(1)\right) \\
&= \sum_{r=1}^{M} \frac{1}{r+1} \sum_{l\in\mathcal{H}_0} \frac{1}{ML}\alpha + \sum_{r=M+1}^{\infty} \frac{1}{r(r+1)} \sum_{l\in\mathcal{H}_0} \frac{1}{L}\alpha + o(1) \\
&= \sum_{l\in\mathcal{H}_0} \frac{\alpha}{ML}\left(\sum_{r=1}^{M} \frac{1}{r+1} + \sum_{r=M+1}^{\infty} \frac{M}{r(r+1)}\right) + o(1) \\
&= \sum_{l\in\mathcal{H}_0} \frac{\alpha}{ML}\left(\sum_{r=1}^{M} \frac{1}{r+1} + \frac{M}{M+1}\right) + o(1) \\
&= \sum_{l\in\mathcal{H}_0} \frac{\alpha}{ML} \cdot \sum_{r=1}^{M} \frac{1}{r} + o(1) \\
&= \sum_{l\in\mathcal{H}_0} \frac{\alpha}{M} + o(1) \\
&\le \alpha + o(1).
\end{aligned}$$

□

Proof of consistency result for thresholded estimator

Proof. We start the proof by decomposing $\|\mathbf{T}_t(\widehat{\boldsymbol{\Theta}}) - \boldsymbol{\Theta}^\star\|_F^2$ as

$$\begin{aligned}
&\|\mathbf{T}_t(\widehat{\boldsymbol{\Theta}}) - \boldsymbol{\Theta}^\star\|_F^2 \\
&= \sum_{\{j,k,s,t\}} (\mathbf{T}_t(\widehat{\Theta}_{jk,st}) - \Theta^\star_{jk,st})^2 \mathbb{1}(|\Theta^\star_{jk,st}| \neq 0) \\
&\quad + \sum_{\{j,k,s,t\}} (\mathbf{T}_t(\widehat{\Theta}_{jk,st}) - \boldsymbol{\Theta}^\star_{jk,st})^2 \mathbb{1}(|\Theta^\star_{jk,st}| = 0).
\end{aligned}$$

By Lemma **??**, we have

$$\begin{aligned}
|\widehat{\Theta}_{jk,st} - \Theta^\star_{jk,st}| &\leq \max_{\{j,k,s,t\}} |\widehat{\Theta}_{jk,st} - \Theta^\star_{jk,st}| \\
&\leq C\sqrt{\frac{\log^4(TN^4)}{T}},
\end{aligned}$$

for some constants C with probability at least $1 - T^{-1}$. Take $t = C\sqrt{\log^4(TN^4)/T}$. When $\Theta^\star_{jk,st} = 0$, $|\widehat{\Theta}_{jk,st}| \leq t$ with probability at least $1 - T^{-1}$.

Then $\|\mathbf{T}_t(\widehat{\Theta}_{jk,st}) - \Theta^\star_{jk,st}\|_F^2$ can be rewritten as

$$\begin{aligned}
&\|\mathbf{T}_t(\widehat{\Theta}_{jk,st}) - \Theta^\star_{jk,st}\|_F^2 \\
&= \sum_{\{j,k,s,t\}} (\widehat{\Theta}_{jk,st} - \Theta^\star_{jk,st})^2 \mathbb{1}(|\Theta^\star_{jk,st}| \neq 0) \\
&\quad + \sum_{\{j,k,s,t\}} (\mathbf{T}_t(\widehat{\Theta}_{jk,st}))^2 \mathbb{1}(|\widehat{\Theta}_{jk,st}| \leq t) \\
&= \sum_{\{j,k,s,t\}} (\widehat{\Theta}_{jk,st} - \Theta^\star_{jk,st})^2 \mathbb{1}(|\Theta^\star_{jk,st}| \neq 0) \\
&\leq \max_{\{j,k,s,t\}} (\widehat{\Theta}_{jk,st} - \Theta^\star_{jk,st})^2 \sum_{\{j,k,s,t\}} \mathbb{1}(|\Theta^\star_{jk,st}| \neq 0) \\
&\leq t^2 m,
\end{aligned}$$

with probability at least $1 - T^{-1}$. □

Some Useful Technical Lemmas

In this section, we will outline some technical lemmas (with proofs) that will be used to prove our main results for consistent estimators.

Lemma 4. For some positive even constant v and some constant $K > 0$,

$$\sum_{v_1+\cdots+v_T=v/2} \prod_{i=1}^{T} \sum_{m=0}^{2v_i} \binom{2v_i}{m} K^{8v_i} 4^{4v_i} (2v_i - m)^{4v_i-2m} \le (Cv^4T)^{v/2},$$

53 for some constant C.

Proof. First, note that

$$\binom{n}{k} \le \left(\frac{ne}{k}\right)^k,$$

then we have

$$\begin{aligned}
&\sum_{v_1+\cdots+v_T=v/2} \prod_{i=1}^{T} \sum_{m=0}^{2v_i} \binom{2v_i}{m} K^{8v_i} 4^{4v_i} (2v_i - m)^{4v_i-2m} \\
&= \sum_{v_1+\cdots+v_T=v/2} \prod_{i=1}^{T} \sum_{m=0}^{2v_i} \binom{2v_i}{2v_i - m} K^{8v_i} 4^{4v_i} (2v_i - m)^{4v_i-2m} \\
&\le \sum_{v_1+\ldots+v_T=v/2} \prod_{i=1}^{T} K^{8v_i} 4^{4v_i} \sum_{m=0}^{2v_i} (2v_i e)^{2v_i-m} (2v_i - m)^{2v_i-m} \\
&\le \sum_{v_1+\ldots+v_T=v/2} \prod_{i=1}^{T} K^{8v_i} 4^{4v_i} \sum_{m=0}^{2v_i} (2v_i e)^{2v_i-m} (2v_i)^{2v_i-m}.
\end{aligned} \tag{S.3}$$

By the sum of a geometric series, we can further simplify (**??**) to

$$\begin{aligned}
&\sum_{v_1+\ldots+v_T=v/2} \prod_{i=1}^{T} K^{8v_i} 4^{4v_i} \frac{(4v_i^2 e)^{2v_i+1} - 1}{(4v_i^2 e) - 1} \\
&\le \sum_{v_1+\ldots+v_T=v/2} \prod_{i=1}^{T} K^{8v_i} 4^{4v_i} \frac{(4v_i^2 e)^{2v_i+1}}{(4v_i^2 e) - 1}.
\end{aligned} \tag{S.4}$$

Since $v_i > 1$, $4v_i^2 e > 2$ and $4v_i^2 e - 1 > 4v_i^2 e/2$, (**??**) can be upper bounded by

$$\begin{aligned}
&\sum_{v_1+\ldots+v_T=v/2} K^{4v} 4^{2v} \prod_{i=1}^{T} (4v_i^2 e)^{2v_i+1} \frac{2}{4v_i^2 e} \\
&= \sum_{v_1+\ldots+v_T=v/2} K^{4v} 4^{2v} 2^T (v^2 e)^v \\
&= \binom{v/2 + T - 1}{v/2} (16K^4 e)^v 2^T v^{2v}
\end{aligned}$$

$$
\begin{aligned}
&\leq (\frac{(v/2+T-1)e}{v/2})^{v/2}(256K^8e^2)^{v/2}(2^{2T/v})^{v/2}(v^4)^{v/2} \\
&\leq (Cv^4T)^{v/2},
\end{aligned}
$$

for some constant C, where the first inequality is obtained by an application of the binomial inequality. □
